# Boundary Conditions Cause Different Generic Bifurcation Structures in Turing Systems

**DOI:** 10.1007/s11538-022-01055-x

**Published:** 2022-08-11

**Authors:** Thomas E. Woolley

**Affiliations:** Cardiff School of Mathematics, Cardiff University, Senghennydd Road, Cardiff, CF24 4AG UK

**Keywords:** Turing instability, Transcritical bifurcation

## Abstract

Turing’s theory of morphogenesis is a generic mechanism to produce spatial patterning from near homogeneity. Although widely studied, we are still able to generate new results by returning to common dogmas. One such widely reported belief is that the Turing bifurcation occurs through a pitchfork bifurcation, which is true under zero-flux boundary conditions. However, under fixed boundary conditions, the Turing bifurcation becomes generically transcritical. We derive these algebraic results through weakly nonlinear analysis and apply them to the Schnakenberg kinetics. We observe that the combination of kinetics and boundary conditions produce their own uncommon boundary complexities that we explore numerically. Overall, this work demonstrates that it is not enough to only consider parameter perturbations in a sensitivity analysis of a specific application. Variations in boundary conditions should also be considered.

## Introduction

Since Turing’s seminal work in 1952 (Turing 1952) on the chemical basis of morphogenesis, the underlying theory of pattern formation, in which a coupled system of partial differential equations (PDEs) is used to describe a reaction–diffusion model, has been applied widely in biology and chemistry (Economou et al. 2012; Kondo and Asai 1996; Sheth et al. 2012; De Kepper et al. 1991; Ouyang and Swinney 1991; Fuseya et al. 2021; Tan et al. 2018; Rudovics et al. 1996) and extended theoretically to include many diverse forms of complexity, such as stochastic interactions, domain growth and spatio-temporal heterogeneity (Cho et al. 2011; Maini et al. 2012; Woolley et al. 2017a, b; Aragón et al. 2012; Krause et al. 2020a). Incredibly, even after 70 years, there is plenty of new research focusing on the theory of diffusion-driven pattern formation and these developments show no signs of slowing (Krause et al. 2021a, b).

With any theory that contends with consistent growth, we must often return to the basics of the theory to ensure that the scaffold of knowledge that we are building rests upon a firm basis and no potential generalisations have been missed (Woolley et al. 2021; Sharpe 2019). In this paper, we return to the most basic components of the Turing bifurcation and demonstrate that it is more complex than the past literature suggests (Maini et al. 2016; Maini and Woolley 2019; Woolley 2014).

Specifically, standard spatially extended linear stability analysis is taught widely to undergraduates and results in an understanding that a specific parameter range (defined as the Turing parameter space) can be generated to satisfy a number of necessary and sufficient conditions that will cause diffusion to drive a stable homogeneous solution to instability, which results in the evolution of the solution away from homogeneity to a stable heterogeneous solution (Murray 2003). Thus, it is common to believe that patterns form if parameters are within a Turing parameter space and that patterns are not seen outside of the Turing parameter space, leading to the criticism that Turing parameter spaces are too small to be biologically relevant (Murray 1982; Bard and Lauder 1974). However, apart from plenty of recent work showing that Turing parameter regions can be made larger than expected (Woolley et al. 2011a, b; Woolley 2011; Woolley et al. 2011c, 2021; Diego et al. 2018; Landge et al. 2020; Vittadello et al. 2021; Scholes et al. 2019), such studies lack nuance as to the higher-order bifurcation structure around the bifurcation point, where subcritical bifurcations can lead to the possibility of patterns appearing outside of the expected range.

It is well known that the Turing instability can appear through a pitchfork bifurcation and that these bifurcations can be subcritical (Leppänen 2004; Benson et al. 1998; Crampin 2000; Dutt 2010, 2012; Grindrod 1996; Nicolis 1995; Auchmuty and Nicolis 1975; Bozzini et al. 2015; Breña-Medina and Champneys 2014; Dalwadi and Pearce 2022). However, what appears to be less well known is that Turing patterns can also stem from a transcritical bifurcation. Critically, any literature that mentions the transcritical bifurcation frequently only mentions it as a passing remark, or as an observation gleaned from numerically derived bifurcation plots (Baurmann et al. 2007; Benson et al. 1998; Jensen et al. 1993; Kouvaris et al. 2015). There appears to have been no rigorously derived form of the transcritical bifurcation and its dependencies on the reaction–diffusion components of the Turing system.

Critically, due to much of the literature using zero-flux boundary conditions, as they are seen as fairly unconstrained boundary conditions (Woolley 2017; Ho et al. 2019; Adamer et al. 2020; Woolley et al. 2012; Winter et al. 2004; Schumacher et al. 2013), it is understandable why these features have been overlooked. Namely, as it will be shown, under zero-flux boundary conditions the bifurcation is of a pitchfork type. However, this does not mean Dirichlet boundary conditions are not important. They are frequently used in combination with French-flag prepatterning systems, or as boundary sources, or sinks (Maini and Myerscough 1997; Jiang et al. 2019). For example, much of the animal digit formation modelling literature requires a source of a signalling protein “Sonic Hedgehog” from a boundary region of the limb bud (Woolley et al. 2014a; Sheth et al. 2012).

As a note on terminology, we are going to be making repetitive use of the terms Dirichlet and Neumann boundary conditions. Generally, Dirichlet boundary conditions define fixed concentration values, whilst Neumann boundary conditions define fixed concentration gradient values. The values that these quantities are fixed at are generally arbitrary. However, we are going to abuse these names slightly to give them a specific meaning. Thus, going forward, Neumann boundary conditions will mean that the concentration has zero-flux at the boundaries. Further, Dirichlet boundary conditions will mean that the concentration is fixed to a spatially homogeneous steady state at the boundaries. Note that we will be assuming that such a state exists as it is a fundamental component of the Turing instability theory, which will be presented in the next section. Moreover, many homogeneous steady states may exist, but we will be focusing on just one that is driven unstable by the inclusion of diffusion.

Critically, the changes in bifurcation structure that are produced by altering the boundary conditions from Neumann to Dirichlet are not observed in the linear analysis, which is where most application papers stop (Woolley et al. 2010; Ho et al. 2019; Cho et al. 2011; Hans et al. 2021). In this paper, we rectify this situation by demonstrating that although the Turing bifurcation is canonically a pitchfork bifurcation under Neumann boundary conditions (van Hecke et al. 1994), the Turing bifurcation is canonically a transcritical bifurcation under Dirichlet boundary conditions. Due to these results being independent of the kinetics chosen, we call these bifurcation structures generic. Specifically, our results hold for any standard two-species coupled system of reaction–diffusion equations, on a finite, non-curved, one-dimensional domain, with constant coefficients that undergo a Turing bifurcation.

In Sect. 2, we first derive the standard necessary conditions for Turing patterning to occur using linear stability theory. We then employ weakly nonlinear perturbation theory (Dutt 2010, 2012; Grindrod 1996) to expand around the Turing bifurcation point and note that the Fourier frequencies that the solution can be expanded into are more restricted in the Dirichlet boundary condition case than when compared to the Neumann boundary condition case. From defining two different inner products for the different cases, we derive the transcritical and pitchfork bifurcation structures under the Dirichlet and Neumann boundary conditions, respectively. In Sect. 3, the theory is put to the test as we compare the analytical bifurcation structure with full nonlinear simulations. Notably, although the simulations and theory match at the bifurcation point, we find that the story is not so clear cut as the bifurcation branches are followed. Penultimately, in Sect. 4, we briefly investigate the specific Turing kinetics we use, known as the Schnakenberg kinetics, as they offer non-standard Turing patterns, as their peak heights are not consistent across the domain. Finally, in Sect. 5 we derive conclusions from this missing bifurcation theory and suggest a stronger role for numerics that investigate boundary condition perturbations within sensitivity analysis.

## Theory

We begin with a minimal system of equations which can present Turing patterns (Maini and Woolley 2019; Murray 2003). Namely, a coupled system of two reaction–diffusion partial differential equations (PDEs) representing the random movement and interactions of two morphogen populations, (*u*(*x*, *t*), *v*(*x*, *t*)), on a finite, one-dimensional, non-curved domain, $\Omega$. The two equations are thus,1where $D_u$ and $D_v$ are positive constants representing the diffusion rate of each population, *i.e.* how fast the populations spread randomly through their domain. The reaction terms, *f* and *g*, are nonlinear kinetics defining how the species influence each other’s growth and decay. To fully close the system’s definition, we need to specify initial and boundary conditions. However, this will be done later.

For brevity, we will make use of vector terminology $\varvec{u}^T(x,t)=(u(x,t),v(x,t))$, where the superscript *T* represents that we are considering the transposed vector. The form of Eq. (1) can, thus, be condensed to

2 $$\begin{aligned} \frac{\partial \varvec{u}}{\partial t}=\frac{1}{L^2}\varvec{D}\frac{\partial ^2 \varvec{u}}{\partial x^2}+\varvec{F}(\varvec{u}), \end{aligned}$$

where the matrix $\varvec{D}$ and vector $\varvec{F}$ are defined appropriately.

The parameter *L* allows us to assume that the PDE system is acting on a non-dimensionalised interval of size $|\Omega |=1$. Specifically, increasing, or decreasing, *L* is equivalent to increasing, or decreasing, the domain size of a fully dimensionalised system. Moreover, if *L* is considered to be slowly changing then it can approximate uniform domain growth, a connection that is explored later in Sect. 3.1 (Crampin and Maini 2001a, b; Crampin 2000; Crampin et al. 1999). Since the patterns formed by Turing systems are well known to be highly dependent on domain size (Murray 2003), we will be using *L* as a bifurcation parameter to ensure that we consider only the first bifurcation point, which is the smallest value of *L* at which a stable spatially homogeneous steady state destabilises causing the solution to evolve to a spatially heterogeneous steady state.

It is standard to think about *u* and *v* as being some general biochemical agents, *e.g.* proteins (Woolley et al. 2017b; Woolley 2014). However, the Turing instability has been applied much more generally, to situations where the populations are themselves cells (Kondo 2005; Woolley et al. 2014b; Woolley 2017). Our approach is application independent and, thus, we do not prescribe the identities of *u* and *v* beyond the fact that they satisfy the two criteria of Turing’s instability: (i) system (1) has at least one spatially homogeneous stable steady state $(u_s,v_s)$ in the absence of diffusion (*i.e.* $D_u=D_v=0$) and (ii) the steady state is driven unstable by the inclusion of diffusion. Further, since we are assuming *u* and *v* represent physical quantities, then this implies that the steady state and the entirety of the solution trajectories lie in the positive (*u*, *v*) quadrant. This assumption can be relaxed for mathematically inclined investigations (Barrio et al. 1999) and is purely used for convenience in this current work.

From this set-up, we can now specify initial conditions. Normally, it is usual to consider small random perturbations about the homogeneous steady state,3where $U_u$ and $U_v$ are sampled from a uniform random distribution $\mathcal {U}([-\sigma ,\sigma ])$ at each point $x\in \Omega$ and $\sigma \ll 1$ (Grimmett and Stirzaker 2001). Note that the vertical lines around the initial conditions, on the right of Eq. (3) show that we are taking the absolute value of the perturbation, ensuring that initial condition is positive. As a sleight modification to the general initial condition definition, we note how boundary conditions are included. Under Dirichlet boundary conditions, we ensure that the boundary values are initially fixed to the steady-state values. For Neumann boundary conditions we ensure that each boundary value is fixed to the same value as their neighbour, providing an approximate zero derivative on the boundary. Critically, these initial conditions will not be smooth as we are using a discretised domain when we solve the system numerically. However, during simulation, after a brief temporal boundary layer, the solutions are seen to converge to smooth solutions that satisfy the equations and boundary conditions.

Sometimes we will want to follow specific bifurcation branches, thus, we will need to choose the initial conditions more carefully. Explicitly, we will see that near the Turing bifurcation point the solutions on the subcritical and supercritical branches are always above and below the steady state, respectively. Thus, if we add a non-negative initial perturbation to the homogeneous steady state then we ensure that the solution is never below the homogeneous steady state within the solution domain, causing the simulation to tend to the subcritical branch solution. Equally, if we add a non-positive perturbation to the homogeneous steady state then we ensure that the solution is never above the homogeneous steady state within the solution domain, causing the simulation to tend to the supercritical branch solution (when stable solutions exist). For clarity, initial conditions will always be stated in the simulation figures when specific forms (beyond randomness) are required.

Note that choosing the initial condition so specifically should not be seen as a problem of “fine-tuning”. We are using the initial conditions to explore the bifurcation space and, thus, understand the possible outcomes a simulation with random initial condition could have.

A further subtlety regarding the initial conditions, boundary conditions and the Turing instability stems from understanding what initial perturbations are possible. Namely, under zero-flux conditions spatially homogeneous perturbations are possible and, the PDE can be simplified to an equivalent ODE by removing spatial terms. The resulting stability analysis on the ODE will then still hold for the full PDE system since a perturbation to the ODE is equivalent to a spatially homogeneous perturbation of the PDE. However, this same equivalence does not exist under fixed boundary conditions, because the PDE requires a spatially heterogeneous perturbation due to the boundaries being fixed at the steady state. There has been recent work to patch this link between PDE systems that have different boundary conditions and their simplified ODE analogues (Klika et al. 2018). Presently, we pragmatically skip over these subtleties, because, at least in the present case, the standard ODE analysis provides solutions that work for the full problem of PDE stability analysis.

It should be noted that Turing’s theory does have a robustness problem, in that vastly different patterned states can be achieved from small changes to the initial condition (Crampin et al. 1999; Maini et al. 2012; Woolley et al. 2011c; Bard and Lauder 1974). Whether this sensitivity is problematic, or not, depends greatly on context (Goodwin et al. 1993). For example, if we are considering animal coat pigmentation formation (*e.g.* on dalmatians (Dougoud et al. 2019)) then this sensitivity to initial conditions is beneficial because it provides a natural method of generating the observed uniqueness of patterns. However, if we are dealing with a situation that requires more exact pattern placement over large spatial scales (*e.g.* long bone growth (Tanaka and Iber 2013)) then additional mechanisms must be included to ensure that an approximately repeatable prototype pattern is maintained despite initial noise (Kondo et al. 2021).

Here, we are going to be primarily interested in the first bifurcation on a small domain. Thus, we can at least guarantee the form of growth of the linearised system, which is seen to be similar to that of the full nonlinear system, at least when close to the bifurcation point (Woolley et al. 2021; Krause et al. 2018, 2020a). The restriction to only one unstable wavelength is one of our primary assumptions that will allow our weakly nonlinear analysis to proceed (Schneider and Uecker 2017; Uecker et al. 2014; Nicolis 1995; Olver 2014a).

Since we will be considering two different sets boundary conditions (Neumann and Dirichlet), we will make the following exposition easier by using two different spatial domains. The reason for this is system (1) is translationally invariant, thus, moving the domain will not change the underlying results, but will allow us to only require cosine Fourier expansions, thereby simplifying the constraints of the boundary conditions. Specifically, we are going to consider the domain $\Omega _D=[-1/2,1/2]$ with Dirichlet boundary conditions,4and the domain $\Omega _N=[0,1]$ with zero-flux, or Neumann, boundary conditions,5The lengths of both domains are $|\Omega |=1$ and, thus, as stated this shift will not influence the underlying bifurcation structure that we are considering. The reason for the shift is that under the Dirichlet boundary conditions and near the Turing bifurcation, we expect that the solution will be an even function about the origin and, thus, the steady states of system (1) can be written in the form6We note that these cosine terms are orthogonal, with respect to the following inner product

7 $$\begin{aligned}&\langle \cos ((2n+1)\pi x),\cos ((2m+1)\pi x)\rangle \nonumber \\&\quad =\int ^{1/2}_{-1/2}\cos ((2n+1)\pi x)\cos ((2m+1)\pi x)\text { d}x =\left\{ \begin{array}{ll} 0 &{} n\ne m, \\ 1/2 &{} m=n, \end{array} \right. \end{aligned}$$

where *n* and *m* are integers. As such we can give the Fourier coefficients, $(u_n,v_n)$, the definite form8Under the insulated domain assumption of the Neumann boundary conditions we use Fourier expansions of the form

9 $$\begin{aligned} h(x)=h_0+\sum ^\infty _{n=1}h_n\cos (n\pi x), \end{aligned}$$

where the inner product is analogously defined to be an integration over [0, 1] and the Fourier coefficients are, thus,

10 $$\begin{aligned} h_n=\left\{ \begin{array}{ll} \int ^1_0 h(x) &{} n=0, \\ 2\langle h(x),\cos (n\pi x)\rangle &{} n\ge 1. \end{array} \right. \end{aligned}$$

Finally, as we are going to make repeated use of multivariable Taylor expansion, we define11to be the partial differential of a general function *h* (where *h* will be either *f* or *g*) with respect to a variable, or series of variables, *a*, evaluated at the steady state. For example, the matrix of first-order derivatives, known as the Jacobian, is

12 $$\begin{aligned} \varvec{J}=\begin{pmatrix} f_u &{} f_v\\ g_u&{} g_v \end{pmatrix}. \end{aligned}$$

Although smooth reaction functions, $\varvec{F}$, can be expanded to any required order, the following analysis only requires an expansion up to third order. Thus, we may expect that the closer the reaction function is to a polynomial of less than third order, the better the Taylor series approximation will work.

### Linear Analysis

As mentioned the Turing condition requires the transition from stability to instability with the inclusion of diffusion. Such a bifurcation is easily encapsulated within the standard approach of linear analysis and, although it has been presented many times (Woolley et al. 2017b; Maini et al. 2016; Murray 2003), we include it for completeness. Critically, the boundary conditions do not influence the results in this case. Elucidating their effects requires higher-order expansions, which are explored in Sects. 2.2.1 and 2.2.2.

To understand the stability features of the steady state we investigate what happens to a general Fourier mode that is a small perturbation away from the steady state (Jones et al. 2009; Evans 2010; Olver 2014b; Bronstein and Lafaille 2002). We consider a trajectory of the form13where $0<\epsilon \ll 1$ and where $k=n\pi$, or $(2n+1)\pi$, with $n\in \mathbb {Z}$, depending on whether we are considering Neumann, or Dirichlet boundary conditions, respectively. For now we continue in terms of *k* because the first bifurcation mode of $n=1$, or $k=\pi$, is applicable in both expansion forms and, thus, the bifurcation point in *L* is not influenced by the boundary conditions.

We substitute perturbation (13) into system (1) and assume that, at least initially, the trajectory remains close to the steady state such that quadratic and higher orders of $\epsilon$ can be ignored. Hence, to leading order we obtain14which can be rearranged to produce the following matrix equation15where $\varvec{I}$ is the $2\times 2$ identity matrix. For Eq. (15) to have a nontrivial answer (*i.e.* $u_{k}$ and $v_{k}$ are not both zero), the multiplication matrix must be singular, which can be ensured if and only if the determinant of the matrix is zero,

16 $$\begin{aligned} 0&=\det \left( \lambda \varvec{I}+\frac{k^2}{L^2}\varvec{D}-\varvec{J}\right) ,\nonumber \\&=\lambda ^2-\lambda \left( f_u+g_v-\frac{k^2}{L^2}(D_u+D_v)\right) +\left( \frac{k^2}{L^2}D_u-f_u\right) \left( \frac{k^2}{L^2}D_v-g_v\right) -g_uf_v. \end{aligned}$$

At this point, we consider two cases: (i) diffusion is absent, which is equivalent to setting $k=0$; and (ii) diffusion is present (*i.e.* $k>0$).

In case (i), Eq. (16) simplifies to

17 $$\begin{aligned} 0=\lambda ^2-\lambda \left( f_u+g_v\right) +f_ug_v-g_uf_v. \end{aligned}$$

The solutions of $\lambda$ can easily be extracted from the quadratic equation (17). However, we do not need to know $\lambda$ exactly to identify the stability of the solution. Explicitly, from the form of the expansion in Eq. (13) if the real part of $\lambda$ is positive, *i.e.* Re$(\lambda )>0$, then the perturbations grow, hence the state is unstable, whilst if Re$(\lambda )<0$ the solution decays back to the steady state and the state is stable. Thus, to extract the sign of $\lambda$ it is easier to use the Routh–Hurwitz stability criterion (Anagnost and Desoer 1991; Routh 1877), which states that the steady state is stable if and only if the coefficients of $\lambda$ and the constant term of Eq. (17) are both positive,

18 $$\begin{aligned} f_u+g_v<&0, \end{aligned}$$

19 $$\begin{aligned} f_ug_v-g_uf_v&>0. \end{aligned}$$

In case (ii), we see from Eq. (16) that the coefficient of $\lambda$ must be positive because $k^2, L^2, D_u, D_v>0$ and $f_u+g_v<0$. Thus, the only way to generate an instability (by the Routh–Hurwitz condition) is if the constant term is negative,

20 $$\begin{aligned} \left( \frac{k^2}{L^2}D_u-f_u\right) \left( \frac{k^2}{L^2}D_v-g_v\right) -g_uf_v<0. \end{aligned}$$

For this to be possible, there must be solutions, $k^2$, within the range $k^2_-<k^2<k^2_+$, where

21 $$\begin{aligned} k^2_\pm =L^2\frac{(D_ug_v+f_uD_v)\pm \sqrt{(D_ug_v+f_uD_v)^2-4D_uD_v(f_ug_v-g_uf_v)}}{2D_uD_v}, \end{aligned}$$

thus, requiring

22 $$\begin{aligned} D_ug_v+f_uD_v>2\sqrt{D_uD_v(f_ug_v-g_uf_v)}, \end{aligned}$$

to ensure that $k^2_\pm$ are real and positive. The final requirement we can impose is that we only want the first heterogeneous frequency to be unstable, *i.e.* $k^2_+=\pi ^2$ providing the critical value for *L* at which the bifurcation will occur,

23 $$\begin{aligned} L_c^2=\frac{2D_uD_v\pi ^2}{(D_ug_v+f_uD_v)+\sqrt{(D_ug_v+f_uD_v)^2-4D_uD_v(f_ug_v-g_uf_v)}}. \end{aligned}$$

From these derivations, we can now define the null vector, $\varvec{\Lambda }$, of Eq. (15) when $\lambda =0$ (since we are at the bifurcation point) and $k=\pi$ (since we are considering the first bifurcation). Thus, up to a multiplicative constant,24

### Weakly Nonlinear Analysis

We now perform a weakly nonlinear stability analysis about the uniform steady state (Grindrod 1996; Nicolis 1995; Auchmuty and Nicolis 1975; Bozzini et al. 2015; Wollkind et al. 1994). It should be noted that the weakly nonlinear expansion is an ansatz, one that depends on good evidence from numerical simulation and previous work (Leppänen 2004; Benson et al. 1998; Crampin 2000; Dutt 2010, 2012; Barrass et al. 2006). However, there is still the possibility that the ansatz does not work in a specific case. In such a case it would be interesting to understand why the breakdown occurs. However, such considerations are outside of the current work. We suppose that the bifurcating solutions emerge at $L=L_c$ in a continuous manner, which allows us to expand $\varvec{u}$ in terms of a power series of $0<\epsilon \ll 1$. Note this $\epsilon$ is not the same as the $\epsilon$ used previously, it is simply a small, but nonzero, parameter, which we use to expand both the bifurcation parameter and the solution.

Our initial work is to set up the theory that is independent of the boundary conditions and then include there effects separately. Specifically, in Sect. 2.2.1 we derive the form of the transcritical bifurcation that is driven by the Dirichlet boundary conditions, whilst in Sect.  2.2.2 we derive the form of the transcritical bifurcation that is driven by the Neumann boundary conditions.

At the bifurcation point, after a transient period, during which the stable modes have relaxed exponentially to zero, the solution does not vary with time. By continuity, one expects that for *L* close to $L_c$ the solution, $\varvec{u}$, of the full system will be a slowly varying function of time (Nicolis 1995). This critical slowing down suggests the introduction of new, more relevant time scales (Stanley 1987). Thus, we introduce multiscale perturbation expansions,252627where28Critically, although convergence of this scheme can be guaranteed under the smooth functions and simple boundary conditions we will be considering, we cannot, in general, determine the radius of convergence (Nicolis 1995; Kevorkian and Cole 2012). Also, note that although $\varvec{U}_i$ and $\varvec{u}_i$ are related, they are not to be confused. Specifically, $\varvec{u}_i$ is a Fourier coefficient, whilst $\varvec{U}_i$ is a spatio-temporal function.

Substituting Eqs. (25)–(27) into system (1) and expanding in terms of $\epsilon$ allows us to collect the differing orders of $\epsilon$ into the following cascade of equations:293031where $\varvec{\mathcal {L}}$ is the linear operator

32 $$\begin{aligned} \varvec{\mathcal {L}}=\frac{1}{L_c^2}\varvec{D}\frac{\partial ^2 }{\partial x^2}+\varvec{J}. \end{aligned}$$

Here, we consider only up to third order in $\epsilon$ but in principle any order can be derived. The only limitation is the algebra becomes more cumbersome. Equally, as we will see later, the approximation does not necessarily become more accurate with higher-order approximations (Becherer et al. 2009).

We have already contended with Eq. (29) in Sect. 2.1. Namely, at the bifurcation point of the first possible frequency, $\varvec{U}_1$ is a null vector of Eq. (32). This was derived in Eq. (24), hence, up to a multiplicative constant33where the amplitude function, *a*, is to be determined by a higher-order solvability criterion.

These solvability criteria come from applying the Fredholm alternative theorem (Ramm 2001). Explicitly, since the kernel of $\varvec{\mathcal {L}}$ is nontrivial then so is the kernel of the adjoint operator $\varvec{\mathcal {L}}^T$, which in this case is simply the transposition of $\varvec{\mathcal {L}}$. Suppose $\varvec{w}\in \text {Ker}\left( \varvec{\mathcal {L}}^T\right)$ and consider any equation of the from $\varvec{\mathcal {L}}\varvec{y}=\varvec{z}$ then

$$\begin{aligned} \langle \varvec{w},\varvec{z}\rangle&=\langle \varvec{w},\varvec{\mathcal {L}}\varvec{y}\rangle ,\\&=\langle \varvec{\mathcal {L}}^T\varvec{w},\varvec{y}\rangle ,\\&=0, \end{aligned}$$

where here the inner product is a dot product of the vectors followed by the integrations as defined in Sect. 2. Thus, for $\varvec{\mathcal {L}}\varvec{y}=\varvec{z}$ to have a solution, $\varvec{z}$ must be orthogonal to a basis that spans $\text {Ker}\left( \varvec{\mathcal {L}}^T\right)$. Any terms that are not necessarily orthogonal to the basis (known as secular, or resonant terms) must be manipulated into a form that allows a zero inner product with the basis of $\text {Ker}\left( \varvec{\mathcal {L}}^T\right)$, in turn, this manipulated form provides the solvability criterion.

Since we have $\varvec{\mathcal {L}}$ in Eq. (32) then, explicitly,

34 $$\begin{aligned} \varvec{\mathcal {L}}^T=\frac{1}{L_c^2}\varvec{D}\frac{\partial ^2 }{\partial x^2}+\begin{pmatrix} f_u &{} g_u\\ f_v &{} g_v \end{pmatrix}. \end{aligned}$$

Hence, the kernel of $\varvec{\mathcal {L}}^T$ can be seen to be spanned by35At this point, most other papers tend to focus only on periodic boundary conditions, or zero-flux/Neumann boundary conditions (which are a subsymmetry of the periodic boundary conditions) (Leppänen 2004; Benson et al. 1998; Crampin 2000; Dutt 2010, 2012; Grindrod 1996; Nicolis 1995; Auchmuty and Nicolis 1975; Bozzini et al. 2015). This choice of boundary condition is important because the nonlinear terms of Eq. (30), would all include a function of the form $\cos ^2(\pi x)$. However,

$$\begin{aligned} \cos ^2(\pi x)=\frac{\cos (2\pi x)+1}{2} \end{aligned}$$

and $\cos (\pi x)$ is orthogonal to $\cos (2\pi x)$ and 1 (under the Neumann boundary conditions, see Eqs. (9) and (10)). Thus, if we simply assume that $L_1=0$ and $\varvec{u}_1$ is independent of $t_1$ then the right-hand side of Eq. (30) is orthogonal to Eq. (35). Whence, we must head to the third-order equation (31), if we are to have any hope of generating a solvability criterion that will define the amplitude function, $a(t_1,t_2)$, of Eq. (33). This case is considered in Sect. 2.2.2.

**Fig. 1:**
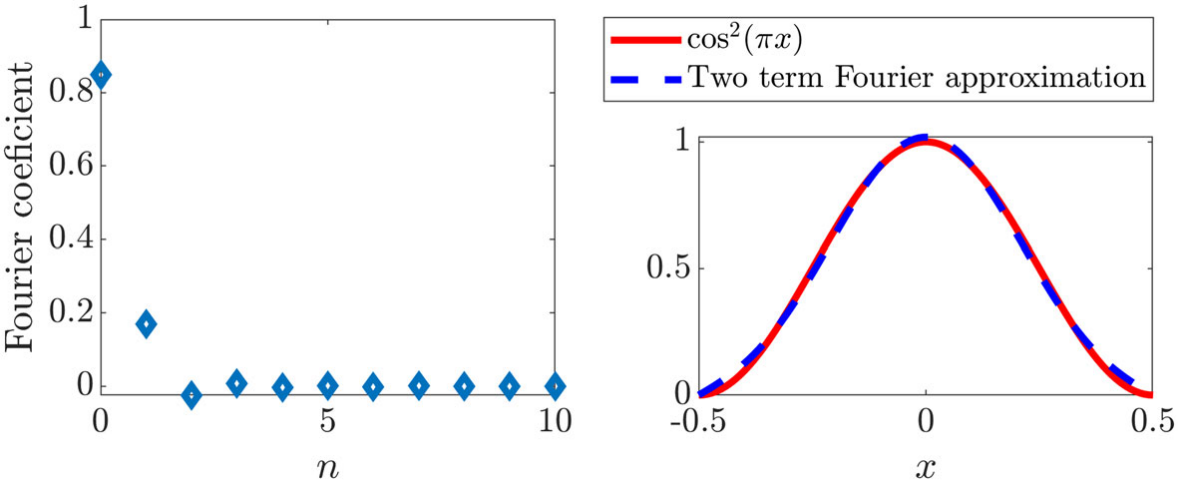
(Colour figure online) Fourier approximation of $\cos ^2(\pi x)$. The left figure presents the value of the Fourier coefficients required to approximate $\cos ^2(\pi x)$. The values are calculated using Eq. (7) and are the explicit values of the coefficients of $\cos ((2n+1)\pi x)$ in Eq. (36). The coefficients are dominated by the first two terms and, as predicted, these two terms provide an excellent approximation to $\cos ^2(\pi x)$ as shown in the right figure

However, in the case of Dirichlet boundary conditions (see Eqs. (6) and (7)) $\cos ^2(\pi x)$ is a secular term. Explicitly

36 $$\begin{aligned} \cos ^2(\pi x)=\sum ^\infty _{n=0}\frac{8(-1)^{n+1}}{\pi (2n+3)(2n+1)(2n-1)}\cos ((2n+1)\pi x) \end{aligned}$$

and, so, the coefficient of $\cos (\pi x)$ is $8/(3\pi )$. We also note that, due to the cubic nature of the denominator in Eq. (36), the first two terms of the expansion provide an excellent approximation to the full solution, see Fig. 1. However, we should highlight that this approximation only holds within the defined solution domain. Outside of $[-0.5,0.5]$ the $\cos ^2(\pi x)$ is no longer well approximated by the Fourier expansion. Of course, we would generally not expect good comparisons outside of this region because the solution domain forms a fundamental part of the inner product definition, through which we derive the Fourier coefficients.

Overall, we deduce that the Dirichlet boundary conditions create resonant terms within the second-order expansion that are not present under Neumann boundary conditions. We explore the resulting solvability criterion stemming from these two sets of boundary conditions in the next two sections.

#### Transcritical Bifurcation Under Dirichlet Boundary Conditions

We focus on Eq. (30) and rewrite it in the explicit formwhere we have substituted $\varvec{U}_1$ from Eq. (33) and rearranged to highlight the $\cos (\pi x)$ and $\cos ^2(\pi x)$ factors. From Eq. (36), we can rewrite

37 $$\begin{aligned} \cos ^2(\pi x)=\frac{8}{3\pi }\cos (\pi x)+NRT, \end{aligned}$$

where *NRT* stands for nonresonant terms. Whence, using Eq. (35), the Fredholm alternative suggests that we must ensure

38 $$\begin{aligned} \left\langle \varvec{\eta },\left( \varvec{C}_1-\frac{8}{3\pi }\varvec{C}_2\right) \cos (\pi x)\right\rangle =0 \end{aligned}$$

for a solution to exist. Evaluating and rearranging Eq. (38) provides the solvability criterion,

39 $$\begin{aligned} \frac{\partial a}{\partial t_1}&=2 \frac{{\pi }^2 \left( \eta \Lambda D_u+D_v \right) a L_1}{{L_c}^3 \left( \eta \Lambda +1 \right) }\nonumber \\&\quad +4 \frac{ \left( \eta {\Lambda }^2 f_{uu} +2 \eta \Lambda f_{uv} + \eta f_{vv}+ g_{uu}\Lambda ^2 +2 g_{uv}\Lambda +g_{vv} \right) a^2 }{3\pi \left( \eta \Lambda +1 \right) }, \end{aligned}$$

which, in turn, can be solved to provide the functional form of the amplitude term, $a(t_1)$. Note that we have suppressed the $t_2$ dependence, which is seen only to influence higher-order approximations and, thus, not defined at this level.

**Fig. 2:**
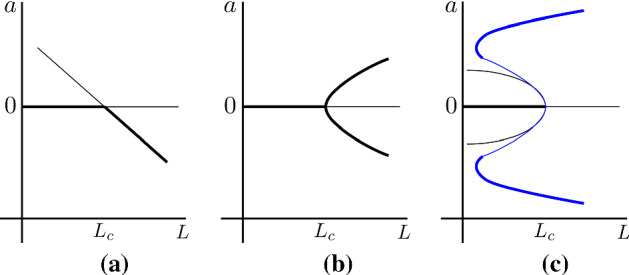
(Colour figure online) Canonical structures of **a** a transcritical bifurcation, **b** a supercritical pitchfork bifurcation and **c** a subcritical pitchfork bifurcation that can arise in a Turing patterning system. The thick lines represent stable amplitude solutions, whilst the thin lines represent unstable amplitude solutions. The additional blue lines in (**c**) illustrate a higher-order approximation of the subcritical pitchfork bifurcation. Specifically, if the expansions are extended to order five in $\epsilon$ we see the curve bends back on itself predicting the existence of stable Turing pattern for $L>L_c$

Although we can fully solve Eq. (39), we are usually interested in the stationary solutions, which we can consider in generality. Equation (39) is a specific form of the more canonical transcritical bifurcation equation,

40 $$\begin{aligned} \frac{\text { d}a}{\text { d}t_1}=p_1 L_1a+p_2a^2, \end{aligned}$$

where the steady states and stability depend on the signs of $L_1$, $p_1$ and $p_2$. Note $L_1=(L-L_c)/\epsilon$ is the bifurcation asymmetry parameter, thus, $L_1<0$ before the bifurcation and $L_1>0$ after the bifurcation. The steady states of Eq. (40) can be seen to be $a=0$ and $-p_1L_1/p_2$. From linear stability analysis, we can see that the stability of these two states switches at $L_1=0$ and that both branches are not stable at the same time. Critically, which branch is stable depends on the signs of $p_1$ and $p_2$. From Sect. 2.1 we know that the homogeneous steady state is locally stable for $L_1<0$ and unstable for $L_1>0$. Thus, due to the transcritical nature of the bifurcation, it must be that alongside the stable branch of inhomogeneous solutions that bifurcate supercritically, there is a branch of unstable heterogeneous solutions that bifurcate subcritically, see Fig. 2a.

Going forward, we assume $p_1$ and $p_2>0$, with the other cases equally easy to contend with. Under these conditions we have that $a=0$ is stable for $L_1<0$ and unstable when $L_1>0$, whereas $a=-p_1L_1/p_2$ is unstable for $L_1<0$ and stable when $L_1>0$. This canonical bifurcation structure is presented in Fig. 2a and compared with the standard supercritical (Fig. 2b) and subcritical (Fig. 2c) pitchfork bifurcations, which are often presented as the only form of Turing bifurcation (Dutt 2010, 2012) and are considered further in Sect. 2.2.2.

The form of the transcritical bifurcation may feel unsatisfactory since the solution cannot follow these straight line approximations for long. Namely, we are assuming that $u>0$ and $v>0$, whereas Fig. 2a suggests that because the amplitude becomes increasingly negative for *L* larger than $L_c$ then the solution $\varvec{u}=\varvec{u}_s+\epsilon \varvec{U}_1$ will eventually go negative. Equally, at first sight our derivation may appear to be limited in its usefulness because for $L<L_c$ the heterogeneous solutions are unstable, leaving only the trivial homogeneous steady state as the expected solution.

To investigate the bifurcation structure further, we extend the analysis to the third-order approximation in $\epsilon$. Specifically, having derived a solution for *a*, we have fully defined $\varvec{U}_1$ through Eq. (33) and, hence, we have fully defined the right-hand side of Eq. (30). Thus, using an expansion of the form41we can fully solve Eq. (30), defining the coefficients in terms of the system parameters and *a*.

Note that the $\alpha$ value in Eq. (41) is completely undetermined because $\varvec{U}_1$ is a null vector of $\mathcal {L}$ (see Eq. (33)). However, we can abuse this freedom to ensure that one of the coefficients of the $\cos (\pi x)$ term is zero. The coefficient $\alpha$ would then need to be specified by the initial conditions, however, since Eq. (41) is an equation for all $\alpha$, we fix it to zero going forward. A further point to note is that because we have to expand $\cos ^2(\pi x)$ in terms of its infinite Fourier cosine series (see Eq. (36)) the form of $\varvec{U}_2$ must also include all of these terms. This is in comparison to the Neumann boundary case of Sect. 2.2.2, where only the first two terms of a Fourier expansion will be needed.

Upon substitution of Eqs. (41) into (30), we can collect and compare terms for every $\cos ((2n+1)\pi x)$ term leading to an infinite set of uncoupled simultaneous equation that can all be solved in closed form after simple, but tedious, manipulation. The explicit forms of $c_{u0}$, $c_{un}$ and $c_{vn}$ are relegated to Appendix A1.

At this point, we will have solutions for both $\varvec{U}_1$ and $\varvec{U}_2$ that can be substituted into Eq. (31). Once again the Fredholm alternative has to be applied to enforce the removal of resonant terms. Finally, this will supply an equation in terms of $\partial a/\partial t_2$ and *a*. Key algebraic steps are presented in Appendix A1 and we provide a Maple workbook (Maplesoft, a division of Waterloo Maple Inc.. 2021) of the accompanying algebraic manipulations, which can be found at https://github.com/ThomasEWoolley/Turing_bifurcations.

**Fig. 3:**
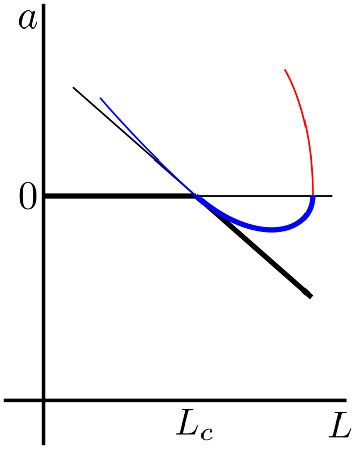
(Colour figure online) Sketch solutions comparing the transcritical bifurcation as derived from Eqs. (40) and (42). Note this is only one form that the branch can take, which is the form most appropriate to our investigations in Sect. 3. Other forms are dictated by the signs of $p_i$ in Eq. (42). We observe that the black lines representing the transcritical bifurcation are complicated by extending the analysis to a third-order expansion. Specifically, the stable supercritical branch is modified to become a quadratic branch that is stable for $a<0$ (blue thick line), linking to an unstable region when $a>0$ (red thin line). The unstable subcritical branch of the third-order expansion (blue thin line), practically matches the second-order expansion (black thin line)

**Fig. 4:**
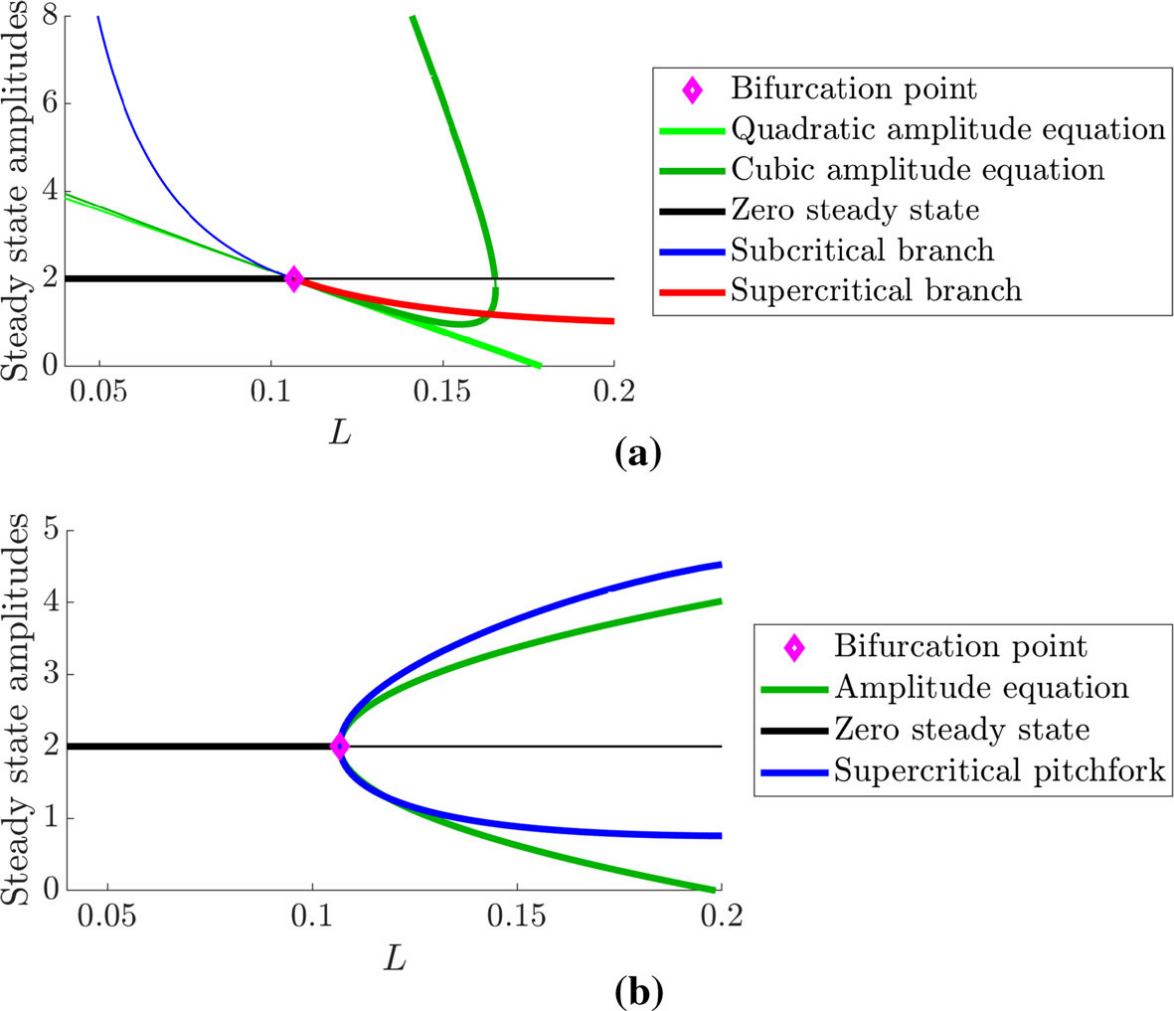
(Colour figure online) Comparing the amplitude Eqs. (49)–(51) with their simulated analogues under **a** Dirichlet boundary conditions and **b** Neumann boundary conditions. In all cases, the diamond point represents the location of $L_c$, the colour represents the method and branch being observed and the thickness of the branch represents the stability of the solutions being observed, *i.e.* thick lines are stable solutions, whilst thin lines are unstable solutions. Further, $u_{s}+\Lambda a$ is plotted in terms of the theoretical derivations and max(*u*) and min(*u*) are plotted in terms of the numerical bifurcation data. In (**a**) we note that the minimum of *u* is the steady state for $L<L_c$, whilst the maximum of *u* is the steady state for $L>L_c$. In (**a**) the light and dark green lines represent the amplitude equations as derived from Eqs. (49) and (50), respectively, which are compared with the pde2path simulations of Eqs. (45) and (46) (blue and red lines). In (**b**), the algebraically derived branch from Eq. (51) (green line) is compared against the blue line obtained from pde2path simulations of Eqs. (45) and (46) (blue line). For the given parameters $L_c\approx 0.11$, $k_+=\pi$ and $k_-\approx 1.02$

Combining the two consistency Eqs. (39) and (A6) together, we can generate a third-order amplitude equation of the form

42 $$\begin{aligned} \frac{\partial a}{\partial t_1}+\epsilon \frac{\partial a}{\partial t_2}=\frac{1}{\epsilon }\frac{\partial a}{\partial t} =\left( p_1(L_1+\epsilon L_2) -p_3\epsilon L_1^2\right) a +(p_2-p_4\epsilon L_1)a^2-\epsilon p_5a^3,\nonumber \\ \end{aligned}$$

where we assume that all of the $p_i>0$ because of the case we are going to consider in Sect. 3. Other sign cases can be investigated just as easily. Here, we comment on the general shape and stability of the solution branches and what further information is provided beyond Eq. (40). Specifically, the cubic nature of Eq. (42) suggests that there is a third branch that has been lost in the lower-order approximation consistency criterion. However, this branch does not bifurcate from $L_c$, rather it could be considered as a bifurcation from infinity that links up with the stable supercritical branch at $L>L_c$. A schematic diagram of the situation is provided in Fig. 3. This schematic diagram can be compared with an actual simulated version in Fig. 4a. Due to this branch not bifurcating close to the point we are expanding around we should be highly sceptical about its existence and indeed it is not seen in the numerical computations later.

The feature that is maybe, at least, qualitatively correct is that the supercritical branch bends upwards to meet the $a=0$ line and then disappears for larger *L* (blue thick line in Fig. 3). Critically, we gain no more information about the structure of the unstable subcritical branch (blue thin line in Fig. 3). Thus, we can conclude that if this branch is to bifurcate further it must do away from the weakly nonlinear regime we are considering and, thus, numerical approaches are more appropriate for such investigations.

Although theoretically trivial to extend the analysis to higher orders, it has actually been shown that even if we are able to generate qualitatively correct bifurcation results, the results do not necessarily get quantitatively better. Indeed, higher-order expansions may not generate any new insights, whilst, at worst, the results may be completely inaccurate (Bozzini et al. 2015; Becherer et al. 2009).

In Sect. 3 we will demonstrate that although the subcritical bifurcation branch starts unstable in the fully nonlinear regime the branch can become stable and, thus, large-amplitude Turing patterns are able to exist for much of $L_1<0$, which is unexpected. Further, the perturbations that are required to push us out of the stability basin of the homogeneous solution are not large, thus, the patterning region can be thought of being larger than derived. Equally, we will demonstrate that the small amplitude supercritical stable branch does not extend far beyond $L_c$, as suggested by Fig. 3.

#### Pitchfork Bifurcation Under Neumann Boundary Conditions

As mentioned, the standard derivation of the Turing amplitude equations tends to produce a canonical pitchfork bifurcation (Grindrod 1996; Nicolis 1995; Auchmuty and Nicolis 1975; Bozzini et al. 2015; Wollkind et al. 1994). We include this derivation for completeness and to see how the situation of Neumann boundary conditions compares with the derivation under Dirichlet boundary conditions.

We return to our comments at the end of Sect. 2.2, where we noted that if we set $L_1=0$ and assume that $\varvec{u}_1$ does not depend on $t_1$ (only $t_2$) then the right-hand side of Eq. (30) will be a function of $\cos ^2(\pi x)$, which is naturally orthogonal to $\cos (\pi x)$, under the inner product for Neumann boundary conditions, as defined in Sect. 2. Thus, Eq. (30) will have an infinite family of solutions of the form43where $\alpha _1$ is, once again, arbitrary due to $\varvec{U}_1$ being in the kernel of $\mathcal {L}$ and, so, is set to zero for simplicity. We substitute Eqs. (33) and (43) into Eq. (30) and collect coefficients of the $\cos (2\pi x)$ and constant terms together. This will lead to four equations that are solved simultaneously to provide the four unknowns. The algebraic forms of $c_{u0}, c_{v0}, c_{u2}$ and $c_{v2}$ are relegated to Appendix A2.

Upon substituting the appropriate components, we will find that the right-hand side of Eq. (31) will contain multiples of $\cos (\pi x)$. Thus, for Eq. (31) to have a solution we enforce the Fredholm solvability criterion and state that the right-hand side of Eq. (31) must be orthogonal to $\varvec{\eta }$, from Eq. (35), *i.e.* $\langle \varvec{\eta },\varvec{\mathcal {L}U}_3 \rangle =0$. Once again, the algebra is laborious but we can extract out a relatively simple canonical pitchfork bifurcation for $a(t_2)$ (see Eq. (A12)),

44 $$\begin{aligned} \frac{\partial a}{\partial t_2}=p_3L_2a-p_4a^3, \end{aligned}$$

where the form of $p_3$ and $p_4$ are presented in Appendix A2.

Whether the branches are supercritical, or supercritical, depends on the signs of $p_3$ and $p_4$. The steady states are $a_0=0$ and $a_\pm =\pm \sqrt{p_3L_2/p_4}$ and, like the case of the transcritical bifurcation, the stability of the branches switches at $L_2=0$. Since we know that $a_0$ is stable for $L_2<0$ then it must be that the branches $a_\pm$ are unstable if they bifurcate subcritically ($L_2<0$), or stable if the bifurcate supercritically ($L_2>0$).

Note that since there are two solutions, $a_\pm$, this means that for every heterogeneous solution that exists for $0<L_2$ the solution’s reflection about the homogeneous steady state is also a solution. Explicitly, for $L\gtrsim L_c$ the troughs and peaks of a Turing pattern can be swapped and the solution will still be valid. This is in contrast to the transcritical bifurcation, where there is one stable amplitude and its reflection is not a solution. This lack of reflective symmetry in the Dirichlet boundary condition case may be counter-intuitive because the kinetics are not changed and any solution under our Dirichlet boundary conditions would also satisfy the boundary conditions under reflection about the steady state. However, as seen in Sect. 2.2.1 the introduction of the Dirichlet boundary conditions breaks the symmetry due to quadratic resonant terms appearing in Eq. (30).

The generic pitchfork supercritical structure is illustrated in Fig. 2, where we can compare and contrast its form with the transcritical bifurcation structure. We illustrate both the sub and supercritical forms of the pitchfork bifurcation in Fig. 2b, c. Since we know that the homogeneous solution becomes unstable at $L=L_c$, the subcritical bifurcation form may be surprising at it suggests there are no stable amplitudes for $L>L_c$, as illustrated by the thin black lines in Fig. 2c. However, as before, we can extend the bifurcation analysis to generate a $5{\text {th}}$-order polynomial in *a* (Bozzini et al. 2015), which predicts that the branches should bend back on themselves (thick blue lines in Fig. 2c) generating the required stable solutions.

The stable subcritical patterns that exist in the interval $L<L_c$ produce a patterning hysteresis in the system. Thus, if *L* is increased passed $L_c$ then patterning is guaranteed. If *L* is then reduced whilst in this patterned state the patterns should remain stable for at least some values of $L<L_c$ (Bozzini et al. 2015). Thus, we again highlight that although linear analysis is incredibly powerful, our insights are limited and it is wrong to state that patterns are not possible outside of the linearly derived patterning parameter space (Woolley et al. 2021).

## Schnakenberg Kinetics Example

In the previous section, we saw that nonlinear analysis was able to shed light on the bifurcation structure near the onset of Turing patterning. However, it was also quickly highlighted that these approaches are severely limited, as the intervals of accurate approximation tend to be very small (Becherer et al. 2009). Thus, to support the theoretical insights we turn to numerical bifurcation tracking.

Specifically, we will be using pde2path, version 3.0, which is a computational continuation package written for MATLAB (Uecker et al. 2014; Dohnal et al. 2014; Uecker 2021a; Engelnkemper et al. 2019; MATLAB 2018). The software is specifically designed to provide bifurcation diagrams for PDEs by applying a modified (pseudo-)arclength parametrisation of solution branches to a spatial discretisation of the PDEs. Critically, the software has been optimised to deal with a large number of degrees of freedom, which will arise due to the PDE being turned into a large ODE system, and compounded further if higher spatial dimensions are considered (Uecker 2021b). It should be noted that, since we have turned to numerical continuation techniques, we can only follow bifurcation branches if we they are found to be connected to known branches. Such continuation techniques are seen to be sufficient in the current case, however more recent methods are available, involving deflation techniques, which would be suitable for finding disconnected bifurcation branches (Farrell et al. 2015).

Although, the specific quantitative shape of a bifurcation diagram will depend on the kinetics being used and will, thus, vary on a case-by-case basis depending on whether the kinetics are biologically or mathematically motivated, we have shown that the qualitative structure of the Turing bifurcation point should only depend on the boundary conditions. Specifically, Dirichlet boundary conditions should lead to a transcritical bifurcation, whilst the Neumann boundary conditions should lead pitchfork bifurcation.

Due to the theory being agnostic to the underlying kinetics, we will be applying pde2path to a set of reaction–diffusion equations which, although originally derived by Gierer and Meinhardt (1972), were popularised by Schnakenberg (1979). Note that we are not saying that these kinetics are biologically accurate in any sense, or directly applicable to a specific case. The Schnakenberg system is simply a well-behaved and well-understood set of mathematically motivated, Turing unstable, reaction kinetics that can be used to demonstrate the dependence of its bifurcation structure on the boundary conditions (Winter et al. 2004; Woolley et al. 2010; Adamer et al. 2020).

Further, the system is a generic form of “cross” kinetics, whereby the two morphogen population patterns are out of phase, such that the peaks of one morphogen correspond to the troughs of the other. Explicitly, the equations are

45 $$\begin{aligned} \frac{\partial u}{\partial t}&=\frac{D_u}{L^2}\nabla ^2 u +1-2u+u^2v, \end{aligned}$$

46 $$\begin{aligned} \frac{\partial v}{\partial t}&=\frac{D_v}{L^2}\nabla ^2 v +3-u^2v. \end{aligned}$$

Normally, the kinetic coefficients and diffusion constants are kept as free parameters. However, for simplicity of illustrating the influence of the boundary conditions, we focus on specifying only $L=L_c+L_1\epsilon +L_2\epsilon ^2$ as the bifurcation parameter and unless otherwise stated $D_u=1/1000$ and $D_v=1/10$.

Applying the analysis from Sects. 2.1 and 2.2, we find that the spatially homogeneous steady state is $(u,v)=(2,3/4)$ and that47which are the bifurcation value of *L*, and the null vectors of $\varvec{\mathcal {L}}$ and $\varvec{\mathcal {L}}^T$, respectively (see Eqs. (23), (24), (33) and (35)). Note that we take the negative form of $\varvec{U}_1$ (which is fine because the vector $\varvec{\Lambda }$ is only defined up to a multiplicative constant) to ensure that *a* and its coefficients have the right sign in the following amplitude equations.

Under Dirichlet boundary conditions, the quadratic amplitude equation is

48 $$\begin{aligned} \frac{\partial a}{\partial t_1}= & {} \frac{800}{201 \pi \left( \sqrt{94}-{\frac{1331}{134}} \right) \left( \sqrt{94}-12 \right) \left( \sqrt{94}-10 \right) }\nonumber \\&\times \left( 151 a^2 \left( {\frac{1462}{151}-\sqrt{94}} \right) +122388 {\frac{a L_{1} \left( \sqrt{2}-{\frac{2104 \sqrt{47}}{10199}} \right) }{\sqrt{120-10 \sqrt{94}}}} \right) , \end{aligned}$$

49 $$\begin{aligned}\approx & {} 15.19a^2+13.93 L_1a, \end{aligned}$$

whilst the higher-order derivation provides a cubic equation of the form

50 $$\begin{aligned} \epsilon \frac{\partial a}{\partial t_2}+\frac{\partial a}{\partial t_1}= & {} \frac{1}{\epsilon }\frac{\text { d}a}{\text { d}t} \approx -35.13 \epsilon a^{3}+ \left( 15.19-268.02 \epsilon L_1 \right) a^2\nonumber \\&+ \left( 13.93 (L_1+\epsilon L_2) - 237.69 L_1^2 \epsilon \right) a. \end{aligned}$$

An accompanying Maple worksheet (which can be found at https://github.com/ThomasEWoolley/Turing_bifurcations) provides all the derivation details for these equations and the final approximation of the numerical coefficients to two decimal places.

Under Neumann boundary conditions, the critical domain length and null vectors are the same as stated in line (47). Further, as shown in Sect. 2.2.2, there is no quadratic leading-order equation for *a* only the canonical pitchfork bifurcation equation

51 $$\begin{aligned} \frac{\partial a}{\partial t_2} = -292.52a^3+13.93aL_2. \end{aligned}$$

Having derived the specific amplitude equations of the Schnakenberg system and given reaction coefficients in the next section, we explore the bifurcation structures numerically and compare Eqs. (49)–(51) to their simulated analogues.

### Simulation

Although the bifurcation tracking software pde2path comes with a Schnakenberg example in its tutorial files, the scripts that accompany all of the following simulations can be found at https://github.com/ThomasEWoolley/Turing_bifurcations. Critically, each file starts with defining a large number of continuation parameter definitions, *e.g.* step size length, Newton iteration tolerance, number of components in the spatial discretisation, etc. We do not discuss these more here and invite the interested reader to view the scripts. However, we do assert that all tolerances were reduced and all discretisations increased to ensure that the following results were not dependent on the simulation inputs. The final chosen parameters were then those that provided a balance between simulation accuracy and speed. Later in this section, we will compare full dynamic spatio-temporal simulations of the nonlinear equations with the bifurcation plots. The spatio-temporal simulations were completed using COMSOL Multiphysics 5.1 (Multiphysics 2021) and again the accompanying codes can be found at https://github.com/ThomasEWoolley/Turing_bifurcations.

First, we focus on the initial Turing bifurcation point. Figure 4a, b demonstrates inextricably the key influence of the boundary conditions on the bifurcation structure. Namely, under Dirichlet boundary conditions (Fig. 4a), the Turing bifurcation is canonically transcritical, whilst under Neumann boundary conditions (Fig. 4b), the bifurcation structure is canonically pitchfork.

In both cases of Fig. 4, we see that the homogeneous steady state ($u=2$) is stable for $L<L_c$ and unstable for $L>L_c$. In Fig. 4a, we see that the subcritical branches provide unstable solutions (thin lines), whilst the supercritical branches produce stable solutions (thick lines). Further, although the nonlinear analysis has provided the correct bifurcation structure, we note that the region over which the amplitude equations are valid is extremely small, with the cubic approximation Eq. (50) providing little more information than the quadratic approximation. Indeed, the supercritical branches of the two approximations lie practically on top of one another, whilst the supercritical approximations give the incorrect suggestions that the solution goes negative in the quadratic approximation case (light green line) and is multivalued in the cubic approximation case (dark green line). What is actually seen is that the subcritical bifurcation undergoes a rapid growth as *L* decreases, whilst the supercritical branch asymptotes to a finite but positive value.

In Fig. 4b, we similarly see that the amplitude equations provide the correct bifurcation structure only within a small interval of the bifurcation point. However, the qualitative comparison between the pitchfork bifurcation structure and its weakly nonlinear approximation is perhaps better than its transcritical partner because the branches track each other well until the approximation inevitably provides a negative solution around $L\approx 0.2$.

**Fig. 5:**
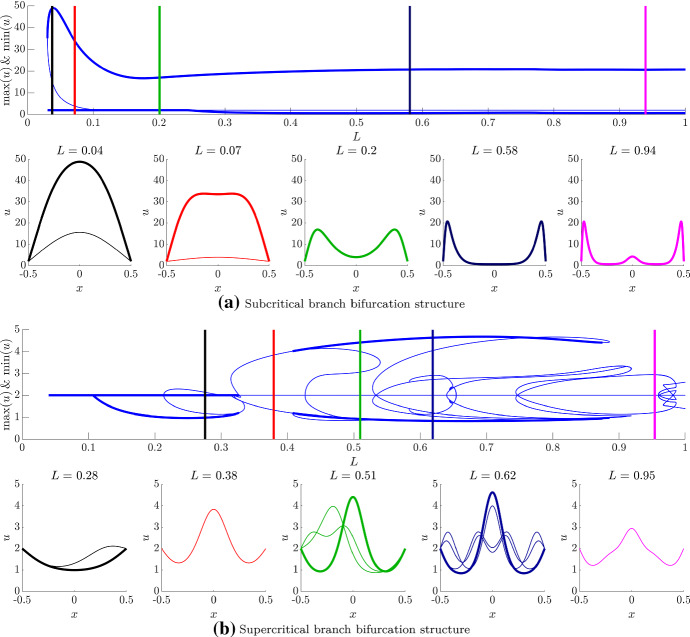
(Colour figure online) Following the bifurcation structure of the **a** subcritical and the **b** supercritical branches of the transcritical bifurcation using pde2path. In each case, the top subfigure is the bifurcation structure of Eqs. (45) and (46) under Dirichlet boundary conditions, as viewed through the maximum and minimum values of *u*, whilst the bottom subfigure presents representative examples of the spatial patterns for different values of *L*. The vertical lines in the top subfigures match the colours of the accompanying simulations and present the location of the *L* parameter being used in the bottom subfigures. In all figures, thick lines represent stable solutions, whilst thin lines represent unstable solutions. Note that multiple patterns plotted on the same axes demonstrate that multiple different patterns are available for the same value of *L*

Seeing that the weakly nonlinear analysis is limited in its abilities we extend our consideration of the bifurcation system through a numerical investigation. In Fig. 5, we track the maximum and minimum values of the *u* population following the subcritical and supercritical branches, separately, over the interval $L\in [0.04,1]$, and demonstrate that even qualitative understanding of the bifurcation structure near the bifurcation point offers limited understanding further away.

Figure 5a presents the bifurcation structure produced by following the subcritical branch of the transcritical bifurcation in the top subfigure and selected solutions in the bottom subfigure. We observe that although the subcritical branch is unstable, this unstable branch folds back on itself producing a multivalued function, providing stable heterogeneous solution for $L<L_c$. Critically, the subcritical branch generates remarkably large-amplitude heterogeneous structures and does so for all $L\gtrsim 0.04$.

These large-amplitude patterns are illustrated along the bottom of Fig. 5a. Here, we have chosen five values of *L* to demonstrate the solutions we would expect to see. The colour of each simulation corresponds to the colour of the line in the top figure, which presents the location of each *L* value of each simulation. Note that the blue line in the top subfigure, which represents the maximum and minimum values of *u*, matches the maximum and minimum values of *u* in the bottom subfigure simulations.

For the left two simulations of the bottom of Fig. 5a, where $L<L_c$ (*i.e.* $L=0.04$ and 0.07) the subfigures show two simulations. The thick lines represent the large-amplitude stable patterns, which would be observed, whilst the thin lines represent the unstable solutions that are found on the subcritical branch.

We observe that as *L* increases from 0.04 the heterogeneous pattern takes on the form of a single peak, whose amplitude grows to around a maximum of $u\approx 50$. This maximum decreases to around $u\approx 20$, where it remains constant. However, observing only the maximum and minimum values does not supply an understanding of what is happening to the pattern across the domain. Specifically, going left to right along the bottom row of Fig. 5a we observe that as the amplitude of the single peak decreases the peak divides evenly into two producing two large-amplitude “bat ears” around a central region that has a fairly low and homogeneous value of *u* (see simulations $L=0.07$–0.58). Note also, that it is during this splitting that the minimum value of *u* first reduces past its steady-state value and stays relatively close to zero from then on.

Finally, as *L* is increased further this central flat region starts to pattern, but with a much smaller amplitude heterogeneity. These patterns are unlike standard Turing structures, which generically show little variation in the maximum and minimum values seen in the peaks and troughs, although such “generic” simulations usually use Neumann boundary conditions. It should be noted that such patterns were investigated in the Schnakenberg model by Ward and Wei (2002) and called “asymmetric spike patterns”. However, these were only considered in a vanishingly small-diffusion-ratio-large-kinetic parameter region and not observed to stem from Dirichlet boundary conditions, but from Neumann boundary conditions.

The existence of stable large-amplitude spatial patterns that stem from the unstable subcritical branch can then be compared with the solutions that stem from the stable supercritical branch. Specifically, as seen in Fig. 5b the supercritical branch only provides stable patterns between the approximate intervals of $[L_c,0.32]$ and [0.41, 0.88]. These two intervals are connected by a region of unstable heterogeneous solutions and all small amplitude solutions are unstable for $L>0.9$.

**Fig. 6:**
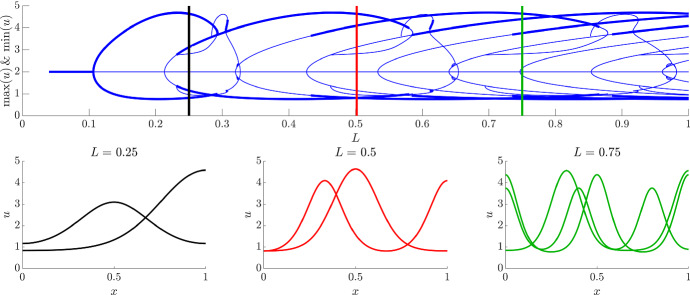
(Colour figure online) Following the bifurcation structure of the pitchfork bifurcation using pde2path. The top subfigure is the bifurcation structure of Eqs. (45) and (46) under Neumann boundary conditions, as viewed though the maximum and minimum values of *u*. The bottom subfigure presents representative examples of the spatial patterns for different values of *L*. The vertical lines in the top subfigures match the colours of the accompanying simulations and present the location of the *L* parameter being used in the bottom subfigures. In the top figure, thick lines represent stable solutions, whilst thin lines represent unstable solutions. For clarity, only the stable solutions have been plotted in the bottom figures. Note that multiple patterns plotted on the same axes demonstrate that multiple different patterns are available for the same value of *L*

The Dirichlet bifurcation structure can be compared with Neumann bifurcation structure, which is in some ways simpler because the symmetry of the initial pitchfork bifurcation means that since we are tracking the maxima and minima of the solution we are following both branches simultaneously. Specifically, the top subfigure of Fig. 6 shows a familiar cascade of higher frequency solutions Crampin et al. (1999); Barrass et al. (2006) moving in and out of stability. For example, if we consider $L=0.25$, the bifurcation structure (black line in Fig. 6) shows that there are two stable and one unstable heterogeneous solutions available. The stable solutions are illustrated in the left simulation on the bottom of Fig. 6. The two stable solutions (represented by the thick black lines) are the $n=1$ and 2 frequency patterns (*i.e.* the pattern has a half peak, or a full peak, respectively).

As *L* increases further more stable and unstable patterns are simultaneously possible. For example, there are four patterns possible when $L=0.5$ (two stable and two unstable) and six patterns possible when $L=0.75$ (three stable and three unstable). Critically, due to solution symmetries there are actually more solutions available. Namely, the spatial reflection of any solution is also possible. Further, any peak in the interior must have a local maximum, *i.e.* a point where $u_x=0$. Thus, because of the Neumann boundary conditions, if the value of the population is the same on the boundaries (effectively generating periodic boundary conditions) then the pattern can be split at any local maximum and rearranged to also provide another solution. For example, consider the stable simulations that have a single interior peak when $L=0.25$ and $L=0.5$. The central peak could be split and the solution could be rearranged to generate a half peak at each boundary and a trough in the centre of the domain. Note this rearranging can only be done with solutions that map to even values of *n*, since odd values of *n* do not satisfy periodic boundary conditions.

Finally, increasing *L* slowly is an approximation of slow domain growth (Maini et al. 2003; Neville et al. 2006; Woolley et al. 2011a, b; Woolley 2011; Woolley et al. 2011c; Krause et al. 2019; Van Gorder et al. 2021). There should also be a dilution term included, but this is small if the domain growth is slow (Barrass et al. 2006; Crampin et al. 2002a, b, 1999; Madzvamuse and Maini 2007). Thus, considering Figs. 5 and 6, we should observe transitions from one stable pattern to another as the domain grows.

Using this correspondence of changing *L* and domain growth, we extend *L* to be the temporally evolving function $L(t)=L_0(1+t/100)$. Thus, the PDEs are now effectively being simulated on a domain that is undergoing uniform linear growth with initial size $L_0$.

**Fig. 7:**
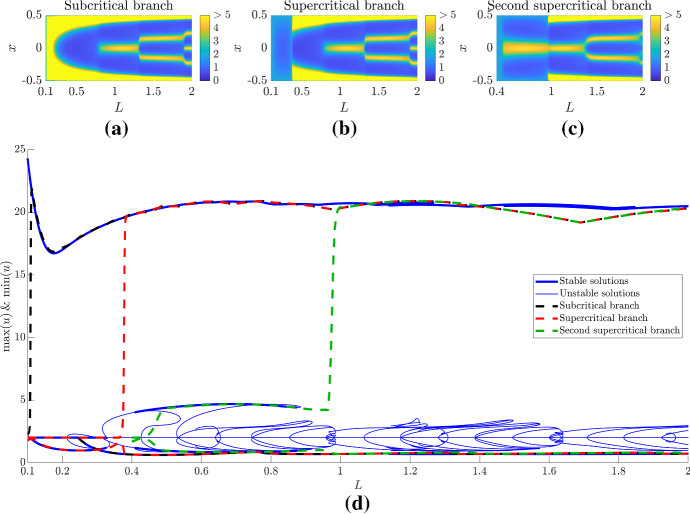
(Colour figure online) Comparing bifurcation diagram and simulations with dynamic *L*. **a**–**c** Illustrating *u* from simulations of Eqs. (45) and (46) with $L=L_0(1+t/100)$ under Dirichlet boundary conditions. Where $L_0=0.1$ in (**a**) and (**b**) simulations and time is run for 1900 time units. In **c**, $L_0=0.4$ and the simulation is run for 400 time units. The initial condition in the left simulation is the steady state plus non-negative perturbations, whilst the initial condition for the middle and right figures is steady state plus non-positive perturbations. Note that because of the large-amplitude boundary peaks we have to saturate the colour bar. Thus, the yellow colour represents all values greater than 5. **d** The maximum and minimum values of *u* are extracted from the simulations in (**a**)–(**c**) (dashed lines) and these are compared with the bifurcation diagram extracted using pde2path (blue lines). The thickness of the bifurcation plot determines the stability of the solution

**Fig. 8:**
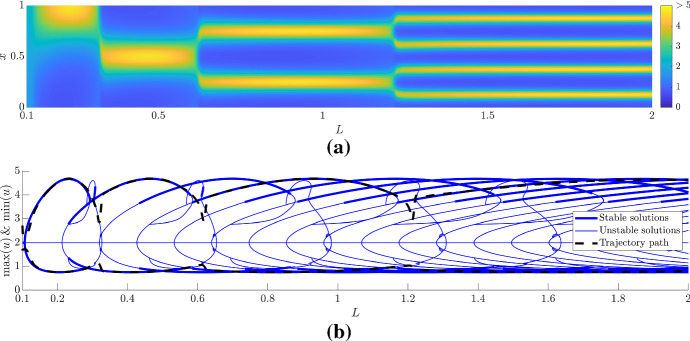
(Colour figure online) Comparing bifurcation diagram and simulations with dynamic *L*. **a** Illustrating *u* from simulations of Eqs. (45) and (46) with $L=L_0(1+t/100)$ under Neumann boundary conditions, where $L_0=0.1$ and time is run for 1900 time units. **b** The maximum and minimum values of *u* are extracted from the simulation in (**a**) (dashed line) and are compared with the bifurcation diagram extracted using pde2path (blue lines). The thickness of the bifurcation plot determines the stability of the solution

In Figs. 7 and 8, we simulate the Schnakenberg kinetics, Eqs. (45) and (46), under the assumption of time dependent *L*, for values of *L* larger than seen previously. Not only do we illustrate the evolving patterns that arise, but we extract the maxima and minima of the solutions and compare these to the bifurcation diagrams.

Firstly, we consider the Dirichlet boundary set-up and start the simulation close to the different solution branches. The simulations of Fig. 7a, b follow the subcritical branch and supercritical branches from the bifurcation point, respectively, see Fig. 7d. To achieve this we start the simulations just before the bifurcation, $L_0=0.1<L_c$ and choose different perturbations. Specifically, the solutions on the subcritical and supercritical branches are always above and below the steady state, respectively. Thus, a non-negative initial perturbation (*i.e.* $u(x,0)=2+(1/2-x)(x+1/2)$) will always cause the simulation to tend to the subcritical branch solution whilst a non-positive perturbation (*i.e.* $u(x,0)=2+(x-1/2)(1/2+x)$) will always cause the simulation to tend to the supercritical branch solution (when the solutions exist). The simulation in Fig. 7(c) was started near the second stable branch that stems from the supercritical branch. This simulation was initiated with $L_0=0.4$ and a non-positive perturbation, $(x-1/2)(1/2+x)$.

As expected, the maximum and minimum trajectories derived from the simulations in Fig. 7a, c closely follow the bifurcation structure seen in Fig. 7d. The solution following the subcritical branch (Fig. 7a and black dashed line in Fig. 7d) present a rapidly rising maximum that stays throughout the whole simulation, which represents the boundary peaks visualised in Fig. 5a. Further, as *L* increases, small internal peaks appear between the two boundary maximal peaks.

The first supercritical branch simulation (Fig. 7b and red dashed line in Fig. 7d) first presents a small amplitude pattern akin to that seen in the bottom left subfigure of Fig. 5b before following the subcritical branch solutions as the first supercritical branch destabilises. Critically, from comparing Fig. 7a, b it appears as though there is a sizeable delay before the patterning appears, when in fact it is simply the solution initially following a different stable path of solutions.

Finally, the second supercritical branch simulation (Fig. 7c and green dashed line in Fig. 7d) presents a small amplitude pattern akin to that seen in the middle subfigure of Fig. 5b before following the subcritical branch solutions as this branch destabilises. Here, we note that in contrast to Fig. 7a, b where the maxima appear on the boundaries first, here the maxima form in the middle first and are maintained as the boundary peaks appear.

Since there are no further stable solutions stemming from the supercritical branch for $L>1$ we see that all simulations follow the same bifurcation trajectories thereafter and the only time the trajectories and the bifurcation branches diverge is when the pattern frequency increases and there is rapid realignment of the pattern. For example around $L\approx 1$ and 1.7 the amplitudes of the trajectories record a dip that is not matched in the bifurcation structure. These dips stem from the fact that *L* is being treated as a dynamic parameter in the simulations rather than as an adiabatic stationary parameter as in the bifurcation analysis. Thus, after these divergences the trajectories do tend to realign with the bifurcation structure as the patterns evolve to their new stable structure.

Considering the Neumann boundary condition situation we reproduce the well-known peak splitting phenomenon seen in much of the domain growth literature (Woolley et al. 2011a, b; Crampin 2000; Crampin et al. 1999), see Fig. 8. Namely, as *L* increases the Turing instability occurs and the pitchfork bifurcation ensures that there is only one potential solution (up to symmetries). As *L* increases further the simulation’s maxima and minima trajectories then follow the bifurcation diagram and particularly follows branches that double the pattern’s frequency because of the underlying symmetries that are present in the reaction–diffusion system (Crampin 2000; Crampin et al. 1999).

Comparing Figs. 7 and 8, we can infer that Neumann boundary conditions make Turing pattern formation more robust than the Dirichlet boundary conditions, at least near the initial Turing bifurcation point. Specifically, regardless of the initial condition, the initial pitchfork bifurcation forces the pattern into a $n=1$ pattern and then growth allows pattern doubling to robustly occur (depending on the form of growth). This is in contrast to the Dirichlet boundary condition situation where two very different solutions are viable because of the transcritical bifurcation. Thus, the initial solution heavily depends on the initial condition. However, if the domain grows large enough then, in at least this case of the Schnakenberg kinetics, the finite numerical evidence we have suggests that all solutions should tend to the same pattern because all small amplitude patterns become unstable. Further, after the patterns have converged to the large-amplitude solutions, growth provides a consistent mechanism of robustly generating higher-order patterns.

## A Brief Diversion into Understanding Boundary Peak Height

The following section presents observations that have arisen through comparing the influence of the different boundary conditions on the patterns. Although we present these features and offer insights as to their dependence we do not explicitly solve the question of how they originate, thus, we offer this section as a starting point for the interested reader. Otherwise, this section can be skipped as it does not add more detail to how the bifurcation structures depend on the boundary conditions.

Presently, we have demonstrated that under Dirichlet boundary conditions it is possible to generate non-standard Turing patterns that have large boundary peaks, but we have yet to investigate their dependence on the equations. Specifically, because the large-amplitude patterns are a boundary effect and, thus, must depend on the spatial components, we consider how the spatial parameters influence the height of the peaks. We focus our attention on the role of $D_v$ and *L* on the height of the peaks, whilst keeping $D_u=10^{-3}$. Note, we only need to consider one of the diffusion rates because non-dimensionalisation shows us that it is the ratio $D_u/D_v$ that is important, rather than each value explicitly.

Firstly, we need to derive the $(L,D_v)$ parameter region from which patterns can emerge. If we consider the auxiliary Eq. (16) of the first mode (*i.e.* $\cos (\pi x)$) then we find that the stability of the homogeneous solution depends on the sign of the real part of $\lambda$ satisfying

52 $$\begin{aligned} 0= & {} \lambda ^2+\frac{((1000 D_v+1)\pi ^2+3000L^2)\lambda }{1000 L^2}\nonumber \\&+\frac{((4-1000D_v) L^2 \pi ^2 +\pi ^4 D_v+8000 L^4)}{1000 L^4}. \end{aligned}$$

**Fig. 9:**
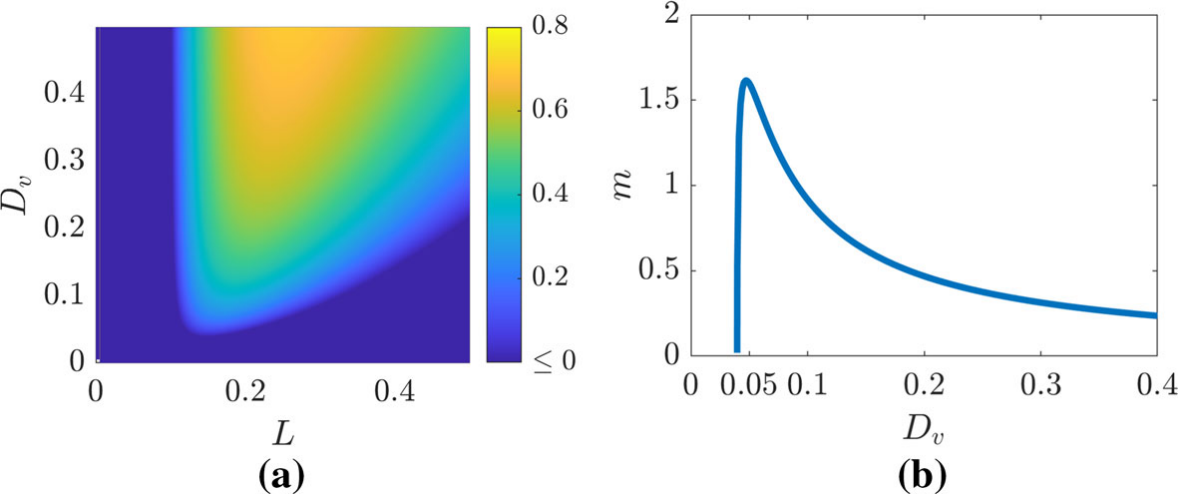
(Colour figure online) Illustrating the Turing unstable parameter region. **a** The $(L,D_v)$ parameter region of Eqs. (45) and (46) where the colour axis illustrates the value of $\lambda$ from Eq. (52). **b** The gradient of the nonzero transcritical bifurcation branch from Eq. (54) with $L=L_c$

The patterning parameter region, *i.e.* where the real part of $\lambda$ is positive is shown in Fig. 9a. The boundary of this region is given by Eq. (52) when $\lambda =0$,

53 $$\begin{aligned} D_v=4{\frac{L^2 \left( 2000 L^2+\pi ^2 \right) }{\pi ^2 \left( 1000 L^2-\pi ^2 \right) }}. \end{aligned}$$

From the explicit form of Eq. (53) we can see that $L\ge \pi /\sqrt{1000}\approx 0.0993$ and $D_v\ge (2+\sqrt{6})(\sqrt{6}+3)\sqrt{6}/1500=D_{vc}\approx 0.0396,$ which occurs when $L=\sqrt{5}\pi \sqrt{2+\sqrt{6}}/100\approx 0.148$, matching the results seen in Fig. 9.

Next, we will show that the boundary peak’s height is related to the steady-state solution of the amplitude equation,

54 $$\begin{aligned} a=-\frac{3\pi ^3(\eta \Lambda D_u+D_v) L_1}{2L_c^3\left( \eta {\Lambda }^2 f_{uu} +2 \eta \Lambda f_{uv} + \eta f_{vv}+ g_{uu}\Lambda ^2 +2 g_{uv}\Lambda +g_{vv} \right) }=:-mL_1.\nonumber \\ \end{aligned}$$

Unfortunately, the dependence of *a* on $D_v$ is not as clear as it first seems from Eq. (54) since $\eta$, $\Lambda$ and $L_c$ also depend on $D_v$. However, we are able to visualise the generic dependence of *m* on $D_v$ in Fig. 9b. We observe a non-monotonic relationship where *a* rapidly rises to a local maximum and then decays to zero as $D_v$ increases.

**Fig. 10:**
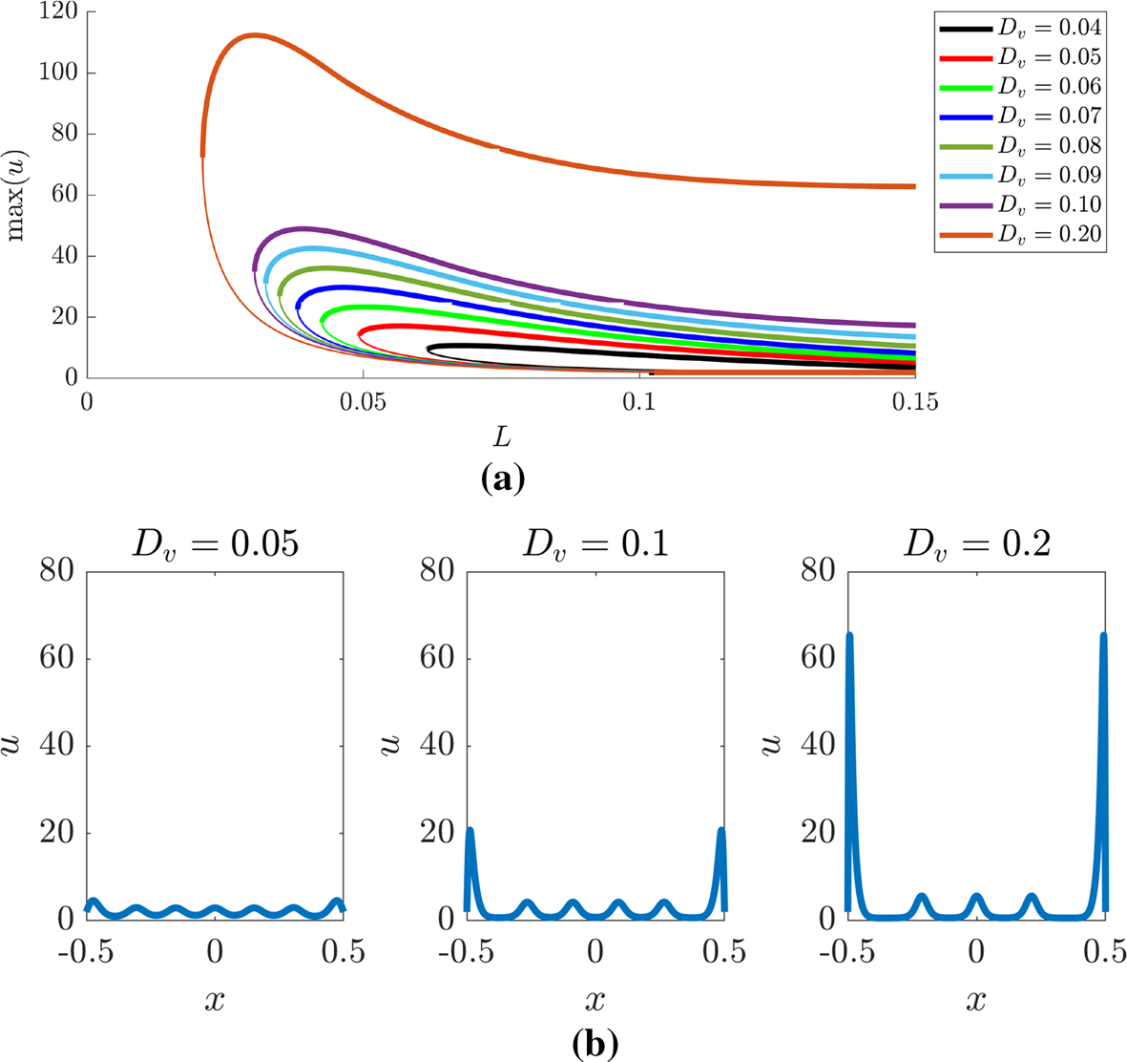
(Colour figure online) Bifurcation diagram and accompanying simulations of Eqs. (45) and (46) over varying values of $D_v$. **a** For all noted values of $D_v$, the subcritical branch of the transcritical Turing bifurcation was followed using pde2path for $L\in [0,0.15]$. The thick lines represent stable solutions, whilst the thin lines represent unstable solutions. **b** Selected simulations of Eqs. (45) and (46) for $D_v=0.05, 0.1$ and 0.2. The simulations were run for 1000 time steps to ensure that they converged to a steady state and the initial condition was a non-negative perturbation of the steady state. In all simulations $L=0.2$

Noting Fig. 9b, we may expect that maximising the coefficient *m* will increase the height of the boundary peaks, as this will cause the transcritical branch gradient to be steeper causing the growth of the transcritical branch to be quicker as *L* decreases from $L_c$, see Fig. 4a. Counterintuitively, it is the opposite that is true, because although increasing *a* is important for a large-amplitude solution, these solutions are only stable once the subcritical branch has folded back on itself, see Fig. 10a. Thus, curves with larger *m* values fold over earlier than curves with smaller values of *m*. Hence, we observe from Fig. 10 that the amplitude of the boundary peaks grows rapidly as $D_v$ increases, whilst leaving the internal peaks relatively unchanged, in terms of height. Moreover, Fig. 10a shows that the range of subcritical patterns grows with increasing $D_v$ to values well below the linear Turing bound of $L\approx 0.0993$ derived above.

## Conclusion

Modelling spatio-temporal phenomena, biological or otherwise, is an incredibly complicated task. Yet despite this complexity, or perhaps because of this, the models and boundary conditions we impose arise from a very small set of recurring structures. For example, if the solution domain is thought to have a constant flux of active population through a boundary then fixed flux boundary conditions can be applied, with zero-flux boundary conditions being the most common, modelling the idea that the populations are not thought to leave their domain of influence. Alternatively, if there is a constant source of population then fixed value boundary conditions can be applied. Of course, boundary condition can be mixed and matched should multiple boundaries exist and, more unusually, we can mix fixed source and flux conditions together to give Robin boundary conditions (Murray 2003). Critically, all of these boundary conditions have analogues in the physical world and, perhaps more importantly, they can now be constructed using techniques from synthetic biology (Krause et al. 2020b; Sheth et al. 2012; Vahey and Fletcher 2014; Dai et al. 2015).

We have demonstrated that boundary conditions can fundamentally alter the bifurcation structure near the Turing bifurcation point of a patterning reaction–diffusion system. Moreover, although the technique of weakly nonlinear analysis is able to highlight this difference the technique may not able to fully characterise the system beyond bifurcation type.

Specifically, although the amplitude equations are able to provide a good understanding of the pitchfork bifurcation in the Neumann boundary condition case the same cannot be said for the Dirichlet boundary condition case. The analysis states that the subcritical branch of the transcritical bifurcation form unstable patterns, whilst the supercritical branch is the one that the simulations will follow. However, numerically, we are able to explore the bifurcation diagram much more freely and discover that although these results hold near the bifurcation point they are not the whole story, with the subcritical branch being much more important to follow, at least in terms of the Schnakenberg kinetics.

Independent of the wider bifurcation structure, we have shown an often missed fact that Turing patterns do not always have to appear through a pitchfork bifurcation. Even among authors who do note the existence of a transcritical bifurcation we have not found anyone who has derived and investigated its structure, even though its structure is simpler than the pitchfork bifurcation, as the system only needs to be expanded to quadratic order, rather than the standard cubic order.

With the freedom to explore the system numerically, we could consider a more general Robin boundary condition and use a parameter to sweep between a scenario that is dominated by the Neumann features, to a scenario that is dominated by the Dirichlet features. Thus, allowing us to investigate how pitchfork bifurcation evolves to a transcritical bifurcation (Dillon et al. 1994). However, preliminary investigations suggest that this is not a trivial matter because it is not a simple transition from one bifurcation type to another, but rather transitioning from Neumann to Robin boundary conditions appears to remove the pitchfork bifurcation leading to the homogeneous steady state becoming stable again. In turn, transitioning from Robin to Dirichlet then allows the transcritical bifurcation to appear. Although potentially tractable delving into such considerations is outside the scope of the current paper and will be left for future work to investigate.

Application of the theory to the Schnakenberg kinetics also led to some unusual results. It is well known that “standard” Turing patterns with consistent peak and trough sizes are common when Neumann boundary conditions are used. However, such standard patterns are the exception, rather than the rule when Dirichlet boundary conditions are used, as small amplitude patterns only occur for a finite parameter region along the supercritical branch. Whereas, the large-amplitude “bat ear” patterns that stem from the unstable subcritical branch provide heterogeneous solutions for an infinitely large $L>L_c$ interval. Further, because of the subcritical nature of the bifurcation the patterning interval extends beyond the region that linear Turing analysis would suggest.

Thus, if Dirichlet boundary conditions are required in an application which presents standard Turing patterns then the system becomes heavily constrained. The parameters and initial conditions would have to be fine-tuned to ensure that the supercritical branch solutions are consistently found. Justifying such constraints and boundary conditions would not be a simple task and would depend heavily on experimental knowledge. Whereas the system is much less constrained under Neumann boundary conditions, which is perhaps why they are so often employed in applications.

Moreover, if the system were applied to a growing domain then it would be virtually impossible to sustain the small amplitude patterns under Dirichlet boundary conditions because the solution trajectory travels through regions of instability and these solutions eventually disappear altogether.

Thus, we realise that just because a system behaves “well” under Neumann boundary conditions, in that the same patterns will always exist under small perturbations of the set-up, allowing many biological applications, this does not mean that Neumann boundary conditions are the correct model of the biological system. Further, since many of the features we have highlighted in this paper are not seen through the standard linear analysis they are not always considered, or appreciated.

Critically, due to the complexity of biological systems it is common for mathematical models to not have their boundary conditions justified strongly, or for justification to be ignored due to reasons of phenomenology (Krause et al. 2021c). However, it should be unsurprising that small changes in boundary conditions can influence the resulting pattern (Arcuri and Murray 1986; Dillon et al. 1994). Moreover, since analytical results can only probe the system close to the bifurcation, which is of limited use (Becherer et al. 2009) we must suggest that if a Turing system is being applied to a biological (or any) application where boundary conditions are unresolved experimentally then the theoretical researchers should include numerical explorations of boundary condition perturbations into their sensitivity analysis, alongside any parameter perturbations. This boundary condition sensitivity analysis will then either: provide further theoretical arguments strengthening the chosen model’s justification; provide interpretation of unexplained biological data; or provide new future avenues of experimental investigation testing model predictions.

## Data Availability

All of the accompanying Matlab, Maple and Comsol codes can be at https://github.com/ThomasEWoolley/Turing_bifurcations.

## Appendix A: Expansion Coefficients

### 1. Dirichlet Boundary Conditions

The weakly nonlinear analysis involves expanding a solution $\varvec{u}$ about the steady in terms of a small parameter, $0<|\epsilon |\ll 1$, *i.e.*

The first function, $\varvec{U}_1$, is seen to be a null vector of the linear operator $\varvec{\mathcal {L}}$, see Eqs. (32) and (33). The second function $\varvec{U}_2$ follows from deriving a consistency condition to define the amplitude function, *a* (Eq. (39)), and substituting $\varvec{U}_1$ into

A1

Finally, assuming $\varvec{U}_2$ has the form

and noting that

$$\begin{aligned} \cos ^2(\pi x)= & {} \sum ^\infty _{n=0}\frac{8(-1)^{n+1}}{\pi (2n+3)(2n+1)(2n-1)}\cos ((2n+1)\pi x)\\= & {} \sum ^\infty _{n=0}c(n)\cos ((2n+1)\pi x), \end{aligned}$$

we can expand Eq. (A1) completely and derive simultaneous equations for the $c_{un}$ and $c_{vn}$ values. Once expanded and rearranged, Eq. (A1) is equivalent to

A2

The solution to which is

A3 $$\begin{aligned} c_{u0}&=\frac{ 4L_c ^2 \left( f_{uu} {\Lambda }^2+2 f_{uv} \Lambda +f_{vv}-{\Lambda }^{3} g_{uu} -2 {\Lambda }^2g_{uv} -\Lambda g_{vv} \right) a^2}{3\pi \left( \eta \Lambda +1 \right) \left( {\pi }^2D_u- f_u L_c ^2 \right) }\nonumber \\&\quad + \frac{2\Lambda {\pi }^2L_1 \left( D_u-D_v \right) a }{L_c \left( \eta \Lambda +1 \right) \left( {\pi }^2D_u- f_u L_c^2 \right) }, \end{aligned}$$

A4 $$\begin{aligned} c_{un}&= {\frac{ \left( \left( - g_v L_c ^{2}+{\pi }^{2}D_v \left( 2n+1 \right) ^{2} \right) \left( {\Lambda }^{2}f_{uu}+2 \Lambda f_{uv}+ f_{vv} \right) + f_v L_c ^{2} \left( g_{uu}{\Lambda }^{2}+2 g_{uv}\Lambda +g_{vv} \right) \right) a ^{2} c\left( n \right) L_c ^{2}}{2 \left( f_u L_c ^{2}-{\pi }^{2}D_u \left( 2n+ 1 \right) ^{2} \right) L_c ^{2}g_v-2 f_v L_c ^{4}g_u-2 {\pi }^{2}D_v \left( 2n+1 \right) ^{2} L_c ^{2}f_u+2 {\pi }^{4} D_u D_v \left( 2n+1 \right) ^{4}}}, \end{aligned}$$

A5 $$\begin{aligned} c_{vn}&=\frac{ \left( \left( - f_u L_c ^{2}+D_u {\pi }^{2} \left( 2n+1 \right) ^{2} \right) \left( {\Lambda }^{2} g_{uu}+2 \Lambda g_{uv}+ g_{vv}\right) + g_u L_c ^{2} \left( f_{uu}{\Lambda }^{2}+2 f_{uv}\Lambda +f_{vv} \right) \right) a ^{2}c\left( n \right) L_c ^{2}}{2 \left( g_v L_c ^{2}-{\pi }^{2}D_v \left( 2n+1 \right) ^{2} \right) L_c ^{2}f_u-2 f_v L_c ^{4}g_u-2 g_v L_c ^{2}D_u {\pi }^{2} \left( 2n+1 \right) ^{2}+2{\pi }^{4}D_u D_v \left( 2n+1 \right) ^{4}}. \end{aligned}$$

We can then substitute the solutions for $\varvec{U}_1$ and $\varvec{U}_2$ into the third-order equation for $\varvec{\mathcal {L}U}_3$ and, once again, use the Fredholm alternative to create a solvability criterion that ensures $\langle \varvec{\mathcal {L}U}_3,\varvec{\eta } \rangle =0$, namely

A6 $$\begin{aligned} \frac{\partial a}{\partial t_2}&={\frac{ \left( \eta f_{uuu}{\Lambda }^{3}+3 \eta f_{uuv}{\Lambda }^{2}+3 \eta f_{uvv}\Lambda +\eta f_{vvv}+g_{uuu}{\Lambda }^{3}+3 g_{uuv}{\Lambda }^{2}+3 g_{uvv}\Lambda +g_{vvv} \right) a^{3}}{8 \left( \eta \Lambda +1\right) }}\nonumber \\&\quad + \left( {\frac{ 8\left( \eta \Lambda f_{uu}+ \eta f_{uv}+ \Lambda g_{uu}+ g_{uv} \right) c_{u0} }{3\pi \left( \eta \Lambda +1 \right) }} - {\frac{ \left( 3{ L_1 }^{2}-2 L_2L_c \right) {\pi }^{2} \left( \eta \Lambda D_u+D_v\right) }{{L_c}^{4} \left( \eta \Lambda +1 \right) }} \right. \nonumber \\&\quad \left. +\sum _{n=1}^\infty \frac{8\left( -1 \right) ^{n+1}\left( \left( \eta \Lambda f_{uu}+\eta f_{uv}+\Lambda g_{uu}+g_{uv} \right) c_{un} +{ \left( \eta \Lambda f_{uv}+\eta f_{vv}+g_{uv}\Lambda +g_{vv} \right) c_{vn} }\right) }{ \left( 2 n+3 \right) \pi \left( \eta \Lambda +1 \right) \left( 4 {n}^{2}-1 \right) } \right) a \nonumber \\&\quad +2 {\frac{\eta {\pi }^{2}D_u L_1 c_{u0} }{{L_c}^{3} \left( \eta \Lambda +1 \right) }}-{\frac{\eta }{\eta \Lambda +1}}\frac{\partial c_{u0}}{\partial t_1}. \end{aligned}$$

Equations (A6) and (39) can then be combined to produce the cubic transcritical bifurcation equation shown in Eq. (42).

### 2. Neumann Boundary Conditions

Section 2.2.2 shows that under Neumann boundary conditions the right-hand side of Eq. (A1) will have no resonant terms to be removed. Thus, the system of equations can be solved directly using a solution of the form

A7

Substituting this into Eq. (A1) leads to linear simultaneous equations, which can be solved to provide

A8 $$\begin{aligned} c_{u0}&={\frac{ \left( \left( {\Lambda }^{2}g_ {uu}+2 \Lambda g_ {uv}+g_ {vv} \right) f_{{v}}-g_{{v}} \left( {\Lambda }^{2}f_ {uu}+2 \Lambda f_ {uv}+f_ {vv} \right) \right) a ^{2}}{4 f_{{u}}g_{{v}}-4 f_{{v}}g_{{u}}}}, \end{aligned}$$

A9 $$\begin{aligned} c_{v0}&=-{\frac{ \left( \left( {\Lambda }^{2}g_ {uu}+2 \Lambda g_ {uv}+g_ {vv} \right) f_{{u}}-g_{{u}} \left( {\Lambda }^{2}f_ {uu}+2 \Lambda f_{uv}+f_ {vv} \right) \right) a ^{2}}{4 f_{{u}}g_{{v}}-4 f_{{v}}g_{{u}}}}, \end{aligned}$$

A10 $$\begin{aligned} c_{u2}&= {\frac{ a^{2}L_c^{2} \left( \left( {\Lambda }^{2}g_{uu}+2 g_{uv}\Lambda +g_{vv} \right) L_c^{2}f_{{v}}+ \left( {\Lambda }^{2}f_{uu}+2 \Lambda f_{uv}+f_{vv} \right) \left( 4 {\pi }^{2}D_v-g_{{v}}L_c^{2} \right) \right) }{-4 f_{{v}}L_c^{4}g_{{u}}+4 \left( 4 {\pi }^{2}D_v-g_{{v}}L_c^{2} \right) \left( 4 {\pi }^{2}D_u-f_{{u}}L_c^{2} \right) }}, \end{aligned}$$

A11 $$\begin{aligned} c_{v2}&= {\frac{ a^{2}L_c^{2} \left( \left( {\Lambda }^{2}f_{uu}+2 \Lambda f_{uv}+f_{vv} \right) L_c^{2}g_{{u}}+ \left( {\Lambda }^{2}g_{uu}+2 \Lambda g_{uv}+g_{vv} \right) \left( 4 {\pi }^{2}D_u-f_{{u}}L_c^{2} \right) \right) }{-4 f_{{v}}L_c^{4}g_{{u}}+4 \left( 4 {\pi }^{2}D_v-g_{{v}}L_c^{2} \right) \left( 4 {\pi }^{2}D_u-f_{{u}}L_c^{2} \right) }}. \end{aligned}$$

Substituting $\varvec{U}_2$ into Eq. (31) will then cause resonant terms to be created that need to be removed through the use of the Fredholm alternative theorem. Thus, applying the requirement that $\langle \varvec{\eta },\varvec{\mathcal {L}U}_3 \rangle =0$ we are able to derive the following form for the time derivative of *a*,

A12 $$\begin{aligned} \frac{\partial a}{\partial t_2}&=2 {\frac{{\pi }^{2} \left( \Lambda D_u g_{{u}}{L_c}^{2}+D_v \left( {\pi }^{2}D_u-f_{{u}}{L_c}^{2} \right) \right) a L_{{2}}}{{L_c}^{3} \left( \Lambda g_{{u}}{L_c}^{2}+{\pi }^{2}D_u-f_{{u}}{L_c}^{2} \right) }} \nonumber \\&\quad +{\frac{ \left( g_{{u}} \left( {\Lambda }^{3}f_{uuu}+3 {\Lambda }^{2}f_{uuv}+3 \Lambda f_{uvv}+f_{vvv} \right) {L_c}^{2}+ \left( {\Lambda }^{3}g_{uuu}+3 {\Lambda }^{2}g_{uuv}+3 \Lambda g_{uvv}+g_{vvv} \right) \left( {\pi }^{2}D_u-f_{{u}}{L_c}^{2} \right) \right) a ^{3}}{8\Lambda g_{{u}}{L_c}^{2}+{\pi }^{2}D_u-f_{{u}}{L_c}^{2}}} \nonumber \\&\quad + {\frac{ {L_c}^{2} \left( \left( \Lambda f_{uu}+f_{uv} \right) \left( { cu}_{{1}} +2c_{u0}\right) + \left( \Lambda f_{uv}+f_{vv} \right) \left( { cv}_{{1}} +2{ cv}_{{0}} \right) \right) g_{{u}}a}{2 \left( \Lambda g_{{u}}{l_{{c}}}^{2}+{\pi }^{2}D_u-f_{{u}}{L_c}^{2} \right) }} \nonumber \\&\quad +{\frac{ \left( {\pi }^{2}D_u-f_{{u}}{L_c}^{2} \right) \left( \left( \Lambda g_{uu}+g_{uv} \right) \left( { cu}_{{1}}+2 { cu}_{{0}} \right) +\left( \Lambda g_{uv}+g_{vv} \right) \left( { cv}_{{1}} +2{ cv}_{{0}} \right) \right) a}{2 \left( \Lambda g_{{u}}{l_{{c}}}^{2}+{\pi }^{2}D_u-f_{{u}}{L_c}^{2} \right) }}. \end{aligned}$$
